# Activation Duty Cycle and Regional Muscle Co-Activation Across GMFCS Levels in Ambulatory Children with Spastic Cerebral Palsy: An Exploratory Surface EMG Study

**DOI:** 10.3390/s26175434

**Published:** 2026-08-27

**Authors:** Peng Huang, Zhen Zhang, Yonggang Tong, Yingfei Sun

**Affiliations:** School of Electronic, Electrical and Communication Engineering, University of Chinese Academy of Sciences, Beijing 101408, China; huangpeng20@mails.ucas.ac.cn (P.H.); zhangzhen221@mails.ucas.ac.cn (Z.Z.); tongyonggang20@mails.ucas.ac.cn (Y.T.)

**Keywords:** cerebral palsy, GMFCS, surface electromyography, muscle co-activation, activation duty cycle, interpretable machine learning, feature association

## Abstract

During self-selected overground walking, we examined participant-level surface electromyography (sEMG) descriptors associated with Gross Motor Function Classification System (GMFCS) levels I–III in 38 ambulatory children with spastic cerebral palsy. Eight-muscle sEMG was summarized using a design-locked pool of 16 peak-normalized envelope and exploratory synergy features. Higher GMFCS level was associated most consistently with longer activation duty cycle, stronger distal and proximal peak-normalized co-activation, and greater inter-muscle spatial entropy. Directions were retained across sensitivity analyses. For secondary within-cohort discrimination, the 16-feature pool yielded a quadratic weighted kappa (QWK) of 0.837±0.046 across 50 repeated participant-level nested cross-validation evaluations. Repeating correlation reduction and four-feature selection inside every outer-training fold yielded QWK 0.849±0.029. Both estimates reuse the 38 children and describe resampling performance, not independent-population generalizability. The core-four specification was post hoc. In an open supplementary workbook containing 195 unique participant IDs, directional concordance was strongest for activation duty cycle and distal co-activation, but direct transfer of the internally fitted 16-feature and core-four models was poor (QWK 0.210 and 0.077). Without healthy controls or measured walking speed and stride length, these cross-sectional results identify descriptors associated with ambulatory GMFCS level, not cerebral-palsy-specific biomarkers or evidence of clinical predictive utility.

## 1. Introduction

Cerebral palsy (CP) comprises permanent disorders of movement and posture that cause activity limitation and arise from non-progressive disturbances in the developing fetal or infant brain [1]. CP is the most common cause of childhood motor disability, and spastic CP is characterized by altered muscle tone and impaired selective motor control [2,3]. Birth prevalence is approximately 1.6 per 1000 live births in high-income settings and is higher in some lower- and middle-income settings [4]. The Gross Motor Function Classification System (GMFCS) stratifies gross motor function into five ordinal levels, with levels I–III denoting ambulatory function of decreasing independence [5]. In routine practice, CP and GMFCS level are established clinically. Because GMFCS summarizes gross motor function rather than muscle activation, quantitative surface electromyography (sEMG) descriptors may complement clinical classification by characterizing neuromuscular coordination across ambulatory levels. Clinical management depends on motor-control impairment, spasticity, contracture, gait pattern, and treatment goals [3,6]. Without a non-CP comparison group, however, within-CP gradients cannot establish CP specificity or a diagnostic biomarker.

sEMG provides a non-invasive window onto muscle activation and inter-muscle coordination. Prior CP studies have examined time–frequency behavior and walking-related fatigue [7,8], and a gait-specific review has described multichannel acquisition, processing, repeatability, and clinical interpretation [9]. CP-focused machine-learning research has addressed heterogeneous tasks, including classification of CP type or GMFCS level from clinical data, prediction of gait events from sEMG, and multimodal wearable-sensor assessment of gait dysfunction [10,11,12]. Other studies have used gait kinematics, entropy measures, time-series deep learning, or reported functional abilities to classify GMFCS level or CP-associated gait patterns [13,14,15,16]. Sensor studies have also compared lower-limb activation across robot-simulated and real walking and evaluated sEMG-based knee-angle estimation [17,18]. Direct gait studies have reported prolonged lower-leg activation and normalization-dependent co-activation estimates, with differences between GMFCS levels I and II under some definitions [19,20,21].

Other sEMG studies have emphasized muscle synergies, co-activation, and selective motor control. Synergy structure and activation have been examined across GMFCS levels and gait-pattern groups [22,23], while reduced synergy complexity has been associated with greater functional impairment [24]. Systematic-review evidence summarizes differences between children with CP and typically developing peers [25], and these outputs depend on EMG filtering and amplitude scaling [26]. Recent work has proposed task-based indices of co-activation, synergy, mirroring, and overflow [27] and situated selective motor control and muscle synergies among the neuromuscular impairments contributing to gait abnormalities in spastic CP [3]. Taken together, this heterogeneity leaves limited directly comparable evidence for participant-level discrimination among ambulatory GMFCS I–III using aggregate eight-muscle sEMG descriptors [2,25,28]. Such descriptors may capture multi-muscle coordination not represented by isolated single-muscle summaries [3,28]. Two gaps are especially relevant: direct comparisons of complementary timing, regional co-activation, and inter-muscle-distribution descriptors within a common eight-muscle protocol remain limited, and the transportability of fitted decision rules across acquisition and feature-generation settings remains uncertain.

**Figure 1:**
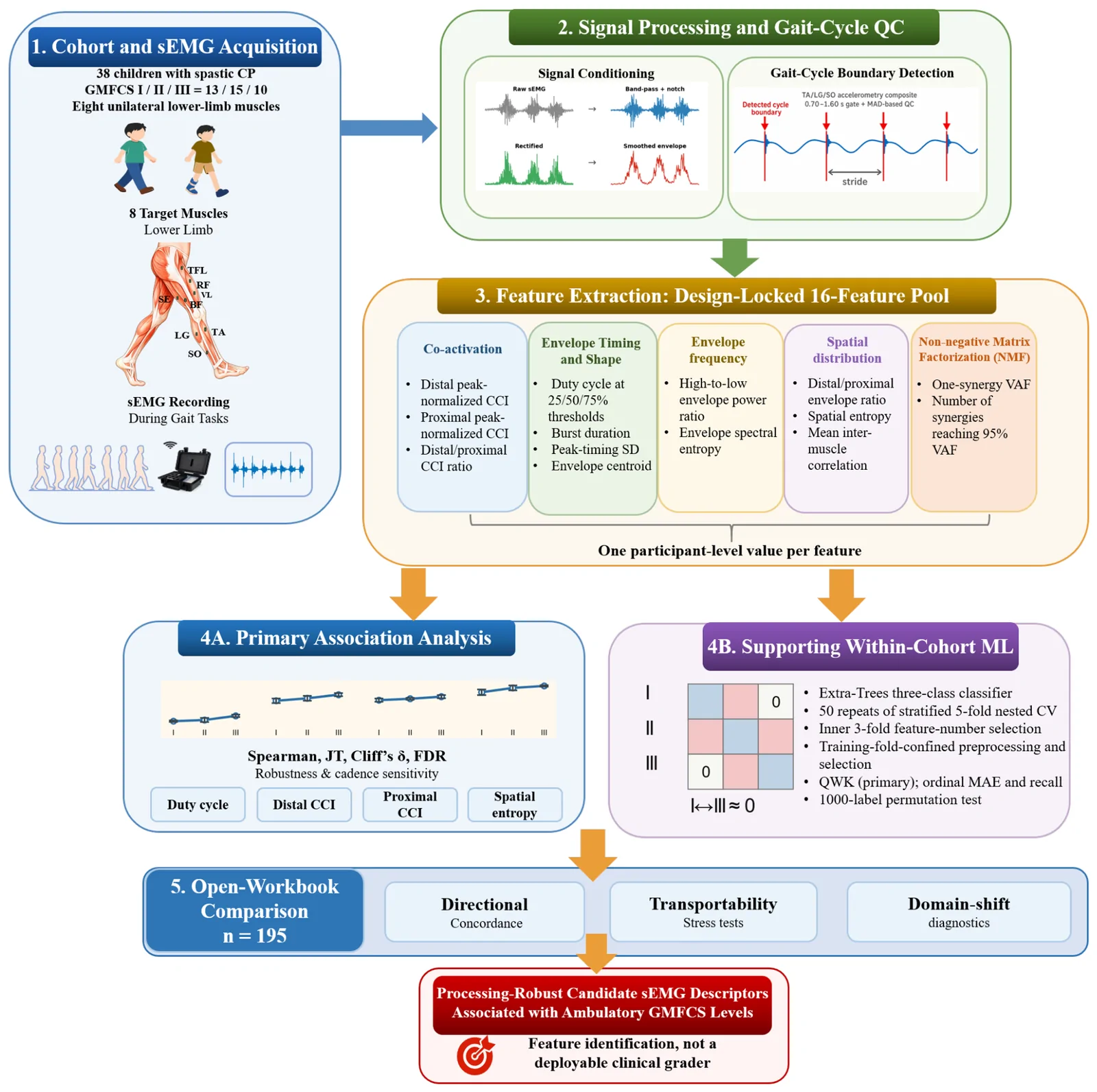
Overview of the study design and analytical workflow. Unilateral surface electromyography (sEMG) was acquired from eight lower-limb muscles in 38 ambulatory children with spastic cerebral palsy (CP), comprising Gross Motor Function Classification System (GMFCS) levels I, II, and III (n=13, n=15, and n=10, respectively). Signal conditioning, gait-cycle quality control, and aggregation produced one feature row per child before cross-validation; within each split, all cycles, muscles, and time points from a child remained in the same partition. A design-locked 16-feature pool was evaluated using primary association analyses and supporting within-cohort machine learning (ML); harmonized features were then compared with an open supplementary workbook (n=195) for directional concordance, transportability stress testing, and domain-shift diagnostics. In Panel 4A, points and error bars denote the mean ± standard deviation across GMFCS levels. Muscle abbreviations: BF, biceps femoris; LG, lateral gastrocnemius; RF, rectus femoris; SE, semitendinosus; SO, soleus; TA, tibialis anterior; TFL, tensor fasciae latae; VL, vastus lateralis. Other abbreviations: CCI, co-activation index; CV, cross-validation; FDR, false discovery rate; JT, Jonckheere–Terpstra; MAD, median absolute deviation; QC, quality control; QWK, quadratic weighted kappa; VAF, variance accounted for.

We therefore used an interpretable, association-first feature strategy. The primary objective was to identify aggregate neuromuscular descriptors associated with GMFCS level and test the stability of their directions. A design-locked participant-level feature pool and prespecified association analyses addressed the first gap; a limited cross-cohort comparison and transportability stress test addressed the second. A design-locked 16-feature pool and leakage-controlled multivariate analysis separately assessed how much ordinal information could be recovered within this cohort under participant-level resampling. This internal analysis was not designed to establish clinical predictive utility. After examination of the complete development cohort, the four leading descriptors formed a post hoc fixed candidate; an outer-fold-contained four-feature analysis evaluated the selection procedure without allowing held-out children to influence selection. Figure 1 summarizes the complete study and analysis workflow.

## 2. Materials and Methods

The workflow comprised data acquisition, gait-cycle boundary detection and quality control, extraction of a design-locked 16-feature pool, feature–GMFCS association analyses, and leakage-controlled feature reduction and repeated nested cross-validation (CV) classification. Additional analyses included an outer-fold-contained four-feature selection sensitivity analysis, a post hoc fixed core-four development-set description, label permutation, cadence and processing sensitivity analyses, and comparison with harmonized features from an open supplementary workbook.

### 2.1. Participants

Thirty-eight ambulatory children with spastic CP participated, distributed across GMFCS levels I (n=13), II (n=15), and III (n=10). Pediatric neurologists diagnosed spastic CP from clinical examination and brain magnetic resonance imaging. Inclusion required spastic CP, ambulatory status (GMFCS I–III), and age 6–12 years; severe cognitive impairment, prior lower-limb orthopedic surgery, and comorbid neuromuscular disorders were exclusion criteria. None of the participants had previously received botulinum toxin injection, oral antispasticity medication such as baclofen, or selective dorsal rhizotomy. Previous rehabilitation or orthotic care was not an exclusion criterion. GMFCS level was assigned independently by two experienced clinicians. Participant characteristics are given in Table 1. Sex and topography were evaluated using two-sided Fisher–Freeman–Halton exact tests, and continuous characteristics using Kruskal–Wallis tests. A cycle-duration-derived cadence proxy was examined as a potential confounder of the leading feature–GMFCS relationships (Section 4.3). The study was approved by the Science and Technology Ethics Committee of the University of Chinese Academy of Sciences (approval number UCASSTEC26-027), and the guardians of all participants provided written informed consent.

### 2.2. Instrumentation and Signal Acquisition

**Figure 2:**
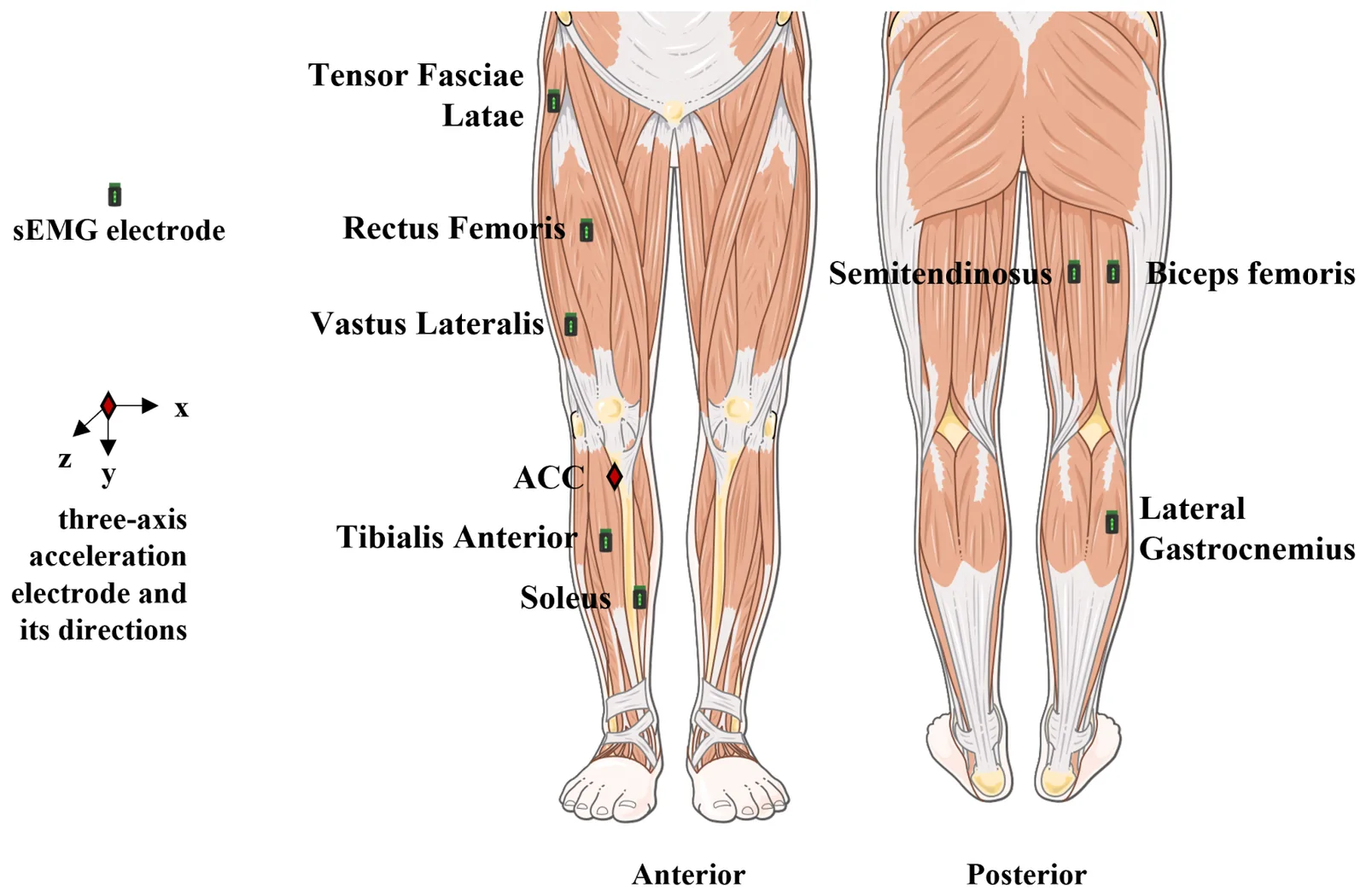
Electrode placement for lower-limb surface electromyography (sEMG) following the Surface Electromyography for the Non-Invasive Assessment of Muscles (SENIAM) guidelines. Gait-cycle boundaries were derived from the triaxial accelerometers embedded in the ankle-region tibialis anterior (TA), lateral gastrocnemius (LG), and soleus (SO) sensors, with the standalone accelerometer (ACC; recorded channel identifier ACC10) as a fallback.

sEMG was recorded from eight lower-limb muscles—tibialis anterior (TA), soleus (SO), lateral gastrocnemius (LG), vastus lateralis (VL), rectus femoris (RF), semitendinosus (SE), biceps femoris (BF), and tensor fasciae latae (TFL)—using the Delsys Trigno™ wireless system at 1000 Hz with built-in 20–450 Hz analog band-pass filtering. Electrode locations and fiber-aligned orientations followed the Surface Electromyography for the Non-Invasive Assessment of Muscles (SENIAM) recommendations [29] (Figure 2). The Delsys Trigno sensors used their fixed silver-bar geometry with a manufacturer-specified 10 mm inter-electrode distance; skin was cleaned with alcohol before application. Children walked at a self-selected speed along a supervised 30 m walkway. The walking protocol did not include instrumented measurements of walking speed or step/stride length. Unilateral sEMG was recorded from the affected side in children with hemiplegia and from the side judged by the examining clinician to have greater clinical involvement in children with diplegia. Fourteen envelope-based descriptors were aggregated across all quality-controlled cycles, whereas the two non-negative matrix factorization (NMF) descriptors were calculated from one deterministically selected cycle per participant. Start-up, deceleration, and turning segments were not explicitly removed. The walking protocol did not distinguish steady-state walking from start-up, deceleration, or turning segments; sensitivity to recording boundaries was therefore evaluated by excluding the first and last retained cycles from each recording (Section 2.17). Gait-cycle boundaries were derived from triaxial accelerometry embedded in the ankle-region sEMG sensors (TA, LG, and SO), with a standalone inertial channel (ACC10) as fallback; detection details are provided in Section 2.4.

### 2.3. Preprocessing

Signals were median-centered and re-filtered offline with a fourth-order Butterworth band-pass (20–450 Hz) and a 50 Hz infinite impulse response (IIR) notch (scipy.signal.iirnotch, quality factor Q=30), applied with zero-phase forward–backward filtering. The signals were then full-wave rectified and smoothed with a 10 ms moving-average window to obtain linear envelopes. Envelopes were segmented into gait cycles and time-normalized to 101 points (0–100% of the cycle). These operations and within-child amplitude normalization did not use GMFCS labels or data from another child. All features in the locked 16-feature pool were computed from these time-normalized envelopes or from their single-cycle NMF representation; absolute raw-EMG amplitude and raw-EMG spectral features were deliberately excluded. All retained cycles, muscles, and time points were collapsed to one participant-level feature row before cross-validation. Within each outer fold, the training and test sets were participant-disjoint, and all cycles from a given child remained within the same partition. Across-participant imputation and every outcome-dependent reduction or selection step were then fitted using training participants only.

### 2.4. Gait-Cycle Boundary Detection and Quality Control

Gait-cycle boundaries were detected from ankle-region accelerometry. For each recording, the triaxial accelerometer signals of the ankle/lower-leg sEMG sensors (TA, LG, and SO) were converted to vector magnitudes, standardized within channel, interpolated to a common time base, and averaged across available ankle-region sensors. The composite trace was band-pass filtered (second-order Butterworth, 0.5–8 Hz, zero phase). Positive and negative peak polarities were evaluated, and the polarity yielding more valid 0.70–1.60 s inter-peak intervals was retained. A within-subject robust duration filter based on the median absolute deviation (MAD) then removed cycles farther than 3.5×1.4826×MAD from the subject median. These criteria may reduce malformed boundary intervals, but they do not identify or exclude start-up, deceleration, or turning cycles. The TA/LG/SO ankle-region composite was available for all 38 recordings. After quality control, stride-time coefficient of variation was 11.50±4.73%, 13.66±6.65%, and 16.97±4.62% for GMFCS I, II, and III, respectively. A cadence proxy used only for confound analysis was calculated as $120/{\bar{T}}_{cycle}$ steps/min; neither cadence nor any gait-timing or quality-control (QC) variable entered the 16-feature model. Candidate and retained cycle counts, removal fractions, and additional boundary-detection details are reported in the Supplementary Materials.

### 2.5. Design-Locked 16-Feature Pool

Before the final 50-repeat evaluation, we locked a pool of 16 participant-level features based on measurement and portability considerations. The pool spanned four complementary domains: regional co-activation, envelope timing and shape, inter-muscle distribution, and exploratory synergy structure. These domains combine established CP-relevant constructs—activation timing, co-activation, and synergy [3,22,27]—with a complementary distributional descriptor, spatial entropy. The pool emphasized aggregate descriptors intended to reduce dependence on absolute sEMG amplitude, device gain, and individual muscles or muscle pairs; local extreme and dispersion summaries were excluded. The resulting pool comprised three regional peak-normalized co-activation descriptors (CCI_peaknorm_distal_mean, CCI_peaknorm_proximal_mean, and CCI_peaknorm_distal_proximal_ratio); six envelope timing/shape descriptors (EnvDuty25_mean, EnvDuty50_mean, EnvDuty75_mean, EnvBurstDuration_mean, EnvPeakTiming_sd, and EnvCentroid_norm_mean); two envelope frequency-distribution descriptors (EnvHL_ratio_high_over_low and EnvSpectralEntropy_mean); three spatial/distribution descriptors (DistalProximalEnvelopeRatio, SpatialEntropy_mean, and EnvIntermuscleCorr_mean); and two single-cycle peak-normalized NMF descriptors (SingleCyclePeakNorm_tVAF1 and SingleCyclePeakNorm_synergies_95VAF). Each child contributed exactly one value per feature: the 14 envelope-based descriptors were aggregated across all quality-controlled cycles, whereas the two NMF descriptors were calculated from one deterministically selected cycle per participant. The resulting analysis matrix therefore contained 38 independent participant rows and 16 feature columns, not one row per cycle, muscle, or time point.

### 2.6. Analysis Roles

The analyses had four distinct roles (Table 2). Feature–GMFCS associations and sensitivity analyses addressed the primary scientific objective. The adaptive Extra-Trees analysis assessed multivariate ordinal information within the internal cohort. An outer-fold-contained post hoc sensitivity analysis estimated the performance of a four-feature selection procedure, with all outcome-dependent selection repeated within each outer-training fold. The named core-four set, chosen only after examination of the complete development cohort, served as a post hoc specification for an external transportability stress test; its internal apparent performance was not treated as an unbiased prediction estimate.

### 2.7. Peak Normalization and Regional Co-Activation

For each child and muscle separately, the linear envelope was divided by the 95th percentile calculated from all retained cycles and all 101 normalized time points for that muscle. Task-based peak amplitude is an established category of reference value when the intended interpretation is relative within-task activation [30]; the 95th percentile was a study-defined operational choice intended to limit the influence of isolated extreme samples, not a percentile prescribed by that consensus source. The normalization reference for a child used no other participant and therefore introduced no cross-subject leakage. Distal muscles were SO, TA, and LG; proximal muscles were VL, RF, SE, BF, and TFL. For each within-region muscle pair, peak-normalized envelope samples from all retained cycles were concatenated and the co-activation index (CCI) was calculated as

(1) $${\mathrm{CCI}}_{ij}=\frac{2\sum_{t}\min\left(E_{i}\left(t\right),E_{j}\left(t\right)\right)}{\sum_{t}E_{i}\left(t\right)+\sum_{t}E_{j}\left(t\right)}.$$

This normalized envelope-overlap measure is conceptually related to established EMG-based co-contraction indices, but it is not identical to the Falconer–Winter or Rudolph formulations, and published indices are not mathematically interchangeable [31,32,33]. Because the present regional summaries include both antagonist and non-antagonist muscle pairs, we refer to the resulting quantities as regional co-activation indices rather than physiological co-contraction measures. The distal and proximal CCI features were the means across all within-region pairs; their ratio summarized the regional balance. Muscle- and subject-specific normalization and aggregation across multiple pairs were intended to reduce sensitivity to common gain and isolated-site variation.

### 2.8. Envelope Shape, Distribution, and NMF Features

Duty-cycle features were the fraction of peak-normalized envelope samples exceeding 0.25, 0.50, or 0.75, calculated per muscle and averaged across all eight muscles. For EnvBurstDuration_mean, a burst was any contiguous run above 0.50 within one muscle and normalized gait cycle; no minimum duration was imposed. Each run length was divided by the 101 samples in that cycle, and the resulting durations were averaged over all detected bursts across muscles and cycles (assigned 0 if none were detected). Peak timing was the within-cycle location of the envelope maximum, expressed from 0 to 1; EnvPeakTiming_sd was its sample standard deviation (SD) across muscles and cycles. For each muscle, all peak-normalized cycles were concatenated and demeaned before Welch power spectral density estimation at a nominal 100 samples per cycle, using a periodic Hann window, nperseg = min(N,256), 50% overlap, constant detrending, and nfft = nperseg. No frequency interpolation was applied. The envelope spectral centroid was calculated within 1–12 cycles^−1^; norm denotes the normalized-envelope input and not an additional division by 12 or by Nyquist. The high-to-low ratio compared power at 6–12 versus 1–3 cycles^−1^, and envelope spectral entropy was calculated over 1–12 cycles^−1^ and averaged over muscles. Spatial entropy quantified the eight-muscle envelope distribution at each concatenated time sample as −$\sum_{m}p_{m}\log\left(p_{m}+10^{-12}\right)/\log8$. Samples with zero total envelope were omitted; the feature was set to missing only if all samples had zero total envelope, and otherwise averaged over retained samples. For inter-muscle correlation, all normalized cycles were concatenated within each muscle, Pearson correlations were calculated for the 28 muscle pairs, and the finite pairwise coefficients were averaged directly; no Fisher-*z* transformation was applied. The distal-to-proximal envelope ratio provided a complementary distribution summary.

For the two synergy descriptors, one gait cycle per child was selected using a pseudorandom generator seeded from the 256-bit Secure Hash Algorithm (SHA-256) digest of the recording identifier. The cycle was peak-normalized by muscle and submitted to NMF with k=1–4 synergies. For each *k*, 20 random initializations were fitted using multiplicative updates with Frobenius loss (maximum 1500 iterations, tolerance $10^{-4}$); restart seeds were deterministically derived from the participant seed, and the solution with the smallest reconstruction sum of squared errors was retained. Variance accounted for (VAF) used the uncentered definition. SingleCyclePeakNorm_tVAF1 is the one-synergy total VAF, and SingleCyclePeakNorm_synergies_95VAF is the smallest *k* reaching VAF ≥0.95 (coded as 5 if none of k=1–4 reached the threshold). Convergence and cycle-sampling sensitivity were evaluated under the same NMF settings across all quality-controlled cycles. Because neither NMF descriptor entered the four leading association descriptors and both depended on a single selected cycle in the locked analysis, NMF was treated as exploratory rather than central predictive evidence.

### 2.9. Feature Reduction, Selection, and Model-Size Sweep

All data-dependent preprocessing was confined to training partitions. The outer evaluation comprised stratified 5-fold cross-validation repeated 50 times, yielding 250 outer folds. Within each outer-training fold, missing values were imputed using feature medians estimated from that fold only. All 16 features were complete for all 38 children. Features were rank-transformed, and the Pearson correlation of feature ranks was used to obtain Spearman correlation. Features connected at |ρ|≥0.8 were grouped into connected components equivalent to single-linkage threshold clusters. Within each cluster, the representative was the feature with the largest training-fold one-way analysis of variance (ANOVA) *F* statistic across the three GMFCS levels, with original column order resolving ties; representatives were ranked by the same criterion. The outer-training fold was then divided by stratified three-fold inner CV, and imputation, correlation clustering, and representative selection were repeated independently within every inner-training split. If $R_{\mathrm{outer}}$ and $R_{j}$ denote the numbers of representatives in the complete outer-training fold and inner-training split *j*, respectively, the largest allowable model size was

(2) $$k_{\max}=\min\left(R_{\mathrm{outer}},R_{1},R_{2},R_{3}\right).$$

Only values in {3,5,8,10} satisfying $k\leq k_{\max}$ were evaluated; thus, only feature counts supported by every relevant training split were considered. For each allowable *k*, the mean validation quadratic weighted kappa (QWK) and its standard error were calculated across the three inner folds. Letting $k_{\mathrm{best}}$ denote the size with the largest mean inner-CV QWK and $s_{k_{\mathrm{best}}}$ denote its standard error, the one-standard-error cutoff was

(3) $$\mathrm{cutoff}={\bar{\mathrm{QWK}}}_{k_{\mathrm{best}}}-s_{k_{\mathrm{best}}}.$$

The smallest allowable *k* reaching this cutoff was selected. On the complete outer-training fold, the top *k* representatives ranked by ANOVA *F* were used to fit the classifier, which predicted the untouched outer-test fold. Outer-test data therefore contributed to neither preprocessing, clustering, feature or representative selection, nor selection of *k*. Selection stability was the proportion of the 250 outer folds in which each feature was selected.

### 2.10. Ordinal Classification and Nested Cross-Validation

GMFCS level was treated as a three-class classification outcome and evaluated using ordinal metrics; nested cross-validation was used to limit selection-induced optimism [34]. The outer evaluation used 50 repetitions of participant-level stratified five-fold CV. Each child appeared once in the test set per repetition and never simultaneously in a training set from the same fold; each outer test fold contained two or three GMFCS-I children, three GMFCS-II children, and two GMFCS-III children. No cycle-level split was performed. Extra-Trees was fixed as the design-locked supporting classifier before the final evaluation and used 500 trees, max_features = ‘sqrt’, min_samples_leaf = 2, no depth limit, and no class weighting. The prespecified comparator set comprised a 500-tree random forest with the same feature and leaf settings; a linear support vector machine (SVM; C=1); a radial basis function (RBF) SVM (C=1, gamma = ‘scale’); and penalized multinomial logistic regression (elastic-net penalty, SAGA solver, $l_{1}$ ratio 0.5, C=0.5, maximum 5000 iterations). A post hoc traditional-model sensitivity benchmark additionally evaluated shrinkage linear discriminant analysis (LDA), *k*-nearest neighbors (kNN; k=5 with distance weighting), Gaussian Naive Bayes, a depth-limited decision tree, gradient boosting, histogram gradient boosting, and AdaBoost. These sensitivity models retained the same outer folds and training-fold-confined correlation reduction, ANOVA ranking, and inner-CV one-standard-error selection of k∈{3,5,8,10}. SVM, logistic-regression, LDA, kNN, and Gaussian-Naive-Bayes pipelines included training-fold standardization. Quadratic discriminant analysis (QDA) was not included because at least one inner-training class had fewer observations than retained features, yielding a rank-deficient class covariance matrix. These estimators were trained as three-class classifiers rather than proportional-odds models; ordinal structure entered evaluation through QWK and ordinal mean absolute error (MAE).

Extra-Trees was prespecified as the primary supporting classifier because its randomized tree ensemble can accommodate possible nonlinear relationships and interactions among aggregate descriptors while randomizing both attribute and cut-point selection [35]. The prespecified random-forest, linear/RBF-SVM, and elastic-net multinomial-logistic-regression comparators were used to assess model-family dependence across the reported repeated-CV evaluation.

No oversampling, undersampling, or synthetic sampling was used. The prespecified primary Extra-Trees model was unweighted. Balanced Extra-Trees was evaluated only as a sensitivity operating point, with all tree parameters unchanged and class_weight = ‘balanced’; within each training partition, class *j* received weight $n_{\mathrm{train}}/\left(3n_{j}\right)$ computed from that partition alone.

The principal classification metric was QWK, with ordinal MAE, accuracy, macro-$F_{1}$, class-specific precision/recall, and I↔III errors as secondary outcomes. “Design-locked” and “fixed before final evaluation” refer to the analytic decision sequence described above. A subject-clustered bootstrap (B=2000) was applied to the existing repeated out-of-fold (OOF) predictions: children were sampled with replacement, and all 50 OOF predictions associated with a sampled child were carried together. These intervals quantify uncertainty conditional on the fitted repeated-CV predictions.

The 50 repetitions changed the train–test assignments but did not create new independent observations: the effective sample size remained 38 children. Accordingly, the repeat-level mean and SD summarize performance and variability under this resampling scheme; a small SD is not evidence of narrow independent-sample uncertainty or external generalizability.

### 2.11. Post Hoc Fixed Four-Feature Analysis

The post hoc fixed four-feature analysis used EnvDuty50_mean, CCI_peaknorm_distal_mean, CCI_peaknorm_proximal_mean, and SpatialEntropy_mean. These descriptors were identified after the full-cohort association and repeated-CV analyses: they were the four most frequently selected features and also defined the principal association result. Because this outcome-informed selection used the complete development cohort, the feature-selection step was not nested, and performance of the named set is reported only as a descriptive post-selection result. The classifier used 500 Extra-Trees, max_features = ‘sqrt’, min_samples_leaf = 2, no depth limit, no class weighting, and no hyperparameter or feature-count selection. All four features were complete; the training-fold median-imputation step therefore did not alter the fixed analysis.

The fixed candidate was evaluated on the same 50 participant-level stratified five-fold partitions as the 16-pool analysis. Because this fixed-feature analysis did not repeat the original full-cohort selection within each training fold, its internal comparisons are reported in Supplementary Section S15b as descriptive post-selection results and are not used to support predictive validity.

### 2.12. Outer-Fold-Contained Four-Feature Selection Procedure

To limit selection-induced optimism, all outcome-dependent selection was repeated separately within every outer-training fold in the four-feature procedure. Within each of the same 250 participant-level outer partitions, training-fold medians were used for imputation, Spearman correlation clustering at |ρ|≥0.8 was fitted on training participants, and the training-fold ANOVA *F* statistic selected one representative per cluster and ranked the representatives. All outer-training folds yielded at least four representatives; the four highest-ranked representatives were used to fit the same fixed Extra-Trees classifier, which then predicted the untouched outer-test participants. Feature identities were allowed to vary across folds. Thus, this analysis estimates the complete four-feature selection procedure rather than the performance of the named post hoc core-four set.

### 2.13. Label Permutation Test

A separate label-permutation analysis used the same one-repeat statistic for the observed and permuted labels: QWK from one stratified 5-fold outer repeat with adaptive one-standard-error *k* selection, distinct from the 50-repeat primary mean. For each of 1000 label permutations, the complete cross-validation procedure—including training-fold median imputation, correlation clustering, ANOVA-based representative selection and ranking, inner-CV adaptive *k* selection restricted to feature counts supported within each training fold, and Extra-Trees fitting—was repeated. The empirical *p* value was calculated as (b+1)/(1000+1), where *b* is the number of null QWK values at least as large as the matched one-repeat observed statistic.

### 2.14. Univariate Analyses

For all 16 features, we reported the GMFCS-level mean ± SD, Spearman association, Kruskal–Wallis and Jonckheere–Terpstra tests, and pairwise Cliff’s δ. Benjamini–Hochberg correction was applied within the 16-feature descriptive family. Participant-level stratified percentile bootstrap intervals (B=10,000; seed 20260724) preserved the observed GMFCS group sizes and quantified 95% uncertainty for Spearman ρ and the GMFCS I-minus-III Cliff’s δ. These post-selection statistics describe magnitude and direction; cross-validated selection frequency summarizes feature-selection stability.

### 2.15. Cadence-Related Sensitivity Analysis

Cadence was not a model input. We examined its association with GMFCS and calculated partial Spearman correlations between each of the four leading association descriptors and GMFCS after rank-transforming and residualizing both variables with respect to the cadence proxy. Pediatric gait research has shown that walking speed affects gait measures broadly [36] and lower-limb sEMG variability specifically [37]. Cadence was treated as a limited proxy because walking speed also depends on step length; direct walking-speed and step/stride-length measurements are needed to quantify speed-related effects.

### 2.16. Topography and Gait-Cycle Quality-Control Sensitivity Analyses

To examine whether the core gradients were driven by the small hemiplegic subgroup, we repeated feature-level descriptive, Spearman, Jonckheere–Terpstra, and GMFCS I-versus-III Cliff’s δ analyses after excluding the five children with hemiplegia. The resulting diplegia-only subset contained 33 children (GMFCS I/II/III =11/14/8); Benjamini–Hochberg false discovery rate (FDR) correction was applied across the four core features within each test family. Separately, we examined retained cycle count, cycle-duration coefficient of variation, and removed-cycle fraction as measured gait-cycle QC quantities. Spearman correlations described their associations with GMFCS and with each core feature. We then rank-transformed and residualized each core feature and GMFCS simultaneously with respect to all three QC quantities and calculated partial Spearman correlations. These analyses assessed sensitivity to topographic composition and the measured gait-cycle QC quantities.

### 2.17. Feature-Definition Sensitivity Analyses

We tested whether the four core feature–GMFCS trends depended on individual processing choices or muscle channels. The features were recomputed using peak-normalization percentiles of 90, 95, and 99; envelope smoothing windows of 5, 10, 20, and 50 ms; gait-cycle duration MAD thresholds of 3.0, 3.5, and 4.0; duration gates of 0.70–1.60 and 0.60–1.80 s; leave-one-muscle-out recomputation for each of the eight muscles; and removal of the most duration-extreme cycle from each recording. As a recording-boundary sensitivity analysis, we additionally removed the first and last chronologically ordered QC-retained cycles from each recording and recomputed the four leading descriptors. This analysis quantifies sensitivity to recording boundaries; it does not identify or exclude start-up, deceleration, or turning cycles. For each variant, we evaluated the Spearman association with GMFCS, the Jonckheere–Terpstra trend with Benjamini–Hochberg correction across the four core features within that variant, and the GMFCS I-versus-III Cliff’s δ. Direction was considered preserved when Spearman ρ>0 and Cliff’s δ<0 under the reported I-minus-III convention.

### 2.18. Open-Workbook Evidence Roles: Directional Comparison, Transportability Stress Tests, and Domain-Shift Diagnostics

The open-workbook analyses comprised (1) directional comparison under approximate harmonization, (2) fixed-model transportability stress tests, and (3) post hoc domain-shift diagnostics. These roles reflect cross-cohort differences in measurement and feature generation.

Harmonized counterparts of the 16 features were constructed from the open workbook (Table S1 accompanying Goudriaan et al.) [23,38]. The analytic workbook contained 195 unique participant IDs with the required inputs (GMFCS I/II/III =108/58/29); the relation to the source article’s n=188 gait-pattern sample and the corresponding exclusion sensitivity analysis are detailed in Supplementary Section S12. Each muscle table contained one row per child with concatenated gait cycles. SO, TA, VL, RF, and BF were aligned directly; LG was mapped to the available gastrocnemius slot, medial hamstrings (MH) to SE as an approximate hamstring mapping, and gluteus medius (GM) to TFL as an anatomically non-equivalent substitution.

The cohorts shared the downstream CCI and envelope equations but differed in acquisition and upstream preprocessing. Internal features were reconstructed from raw Delsys sEMG using project-specific filtering, envelope extraction, gait-cycle boundary detection, resampling, and participant–muscle normalization. The open workbook provided prefiltered, rectified, normalized, and concatenated muscle activations, from which cycles were reconstructed using the available 101-point concatenations. The internal single-cycle peak-normalized NMF quantities were calculated from the internal sEMG data using the stated NMF procedure, whereas the open-workbook tVAF and synergy-count quantities were obtained from the deposited VAF quantities.

For locked-model transportability stress tests, the fixed internal 16-feature Extra-Trees classifier and the core-four candidate were each fitted to all 38 internal participants using 500 trees, max_features = ‘sqrt’, min_samples_leaf = 2, no depth limit, and no class weighting. Each was applied unchanged to the 195 open-workbook participants; any missing-value imputation used internal-cohort medians. Separately, the same specifications were retrained and evaluated within the open-workbook cohort using 50 repetitions of stratified five-fold CV, with and without balanced class weighting. These analyses measured performance after refitting within the open-workbook cohort.

Domain-shift analyses compared marginal feature distributions using two-sample Kolmogorov–Smirnov statistics with Benjamini–Hochberg correction and compared within-cohort Spearman feature–GMFCS associations. Cohort separability was evaluated with repeated-CV logistic-regression and Extra-Trees domain classifiers using either all 16 features or the core four. Alignment by the median and interquartile range (IQR), as well as marginal quantile alignment of the all-16 specification, was evaluated as a post hoc distribution-alignment diagnostic using the complete unlabeled open-workbook feature distribution.

### 2.19. Software and Reproducibility

Analyses were conducted in Python 3.8.3 using NumPy, SciPy, pandas, scikit-learn, and statsmodels; fixed random seeds were used for all stochastic analyses.

## 3. Results

### 3.1. Leading Feature–GMFCS Associations

**Figure 3:**
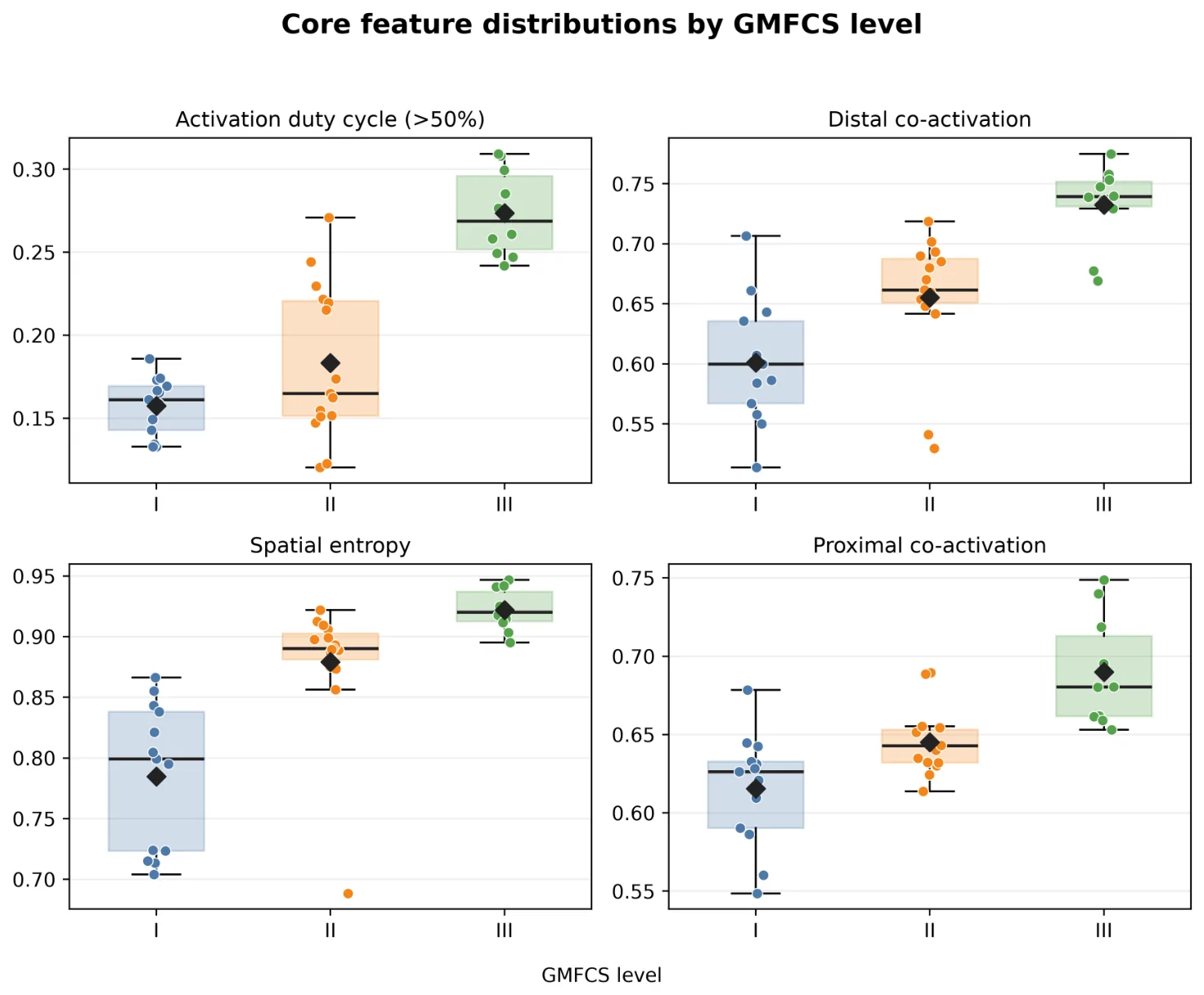
Internal-cohort distributions of the four leading association descriptors—activation duty cycle, distal peak-normalized co-activation, spatial entropy, and proximal peak-normalized co-activation—across GMFCS levels. Points denote children, boxes show the interquartile range and median, and black diamonds denote group means. GMFCS, Gross Motor Function Classification System.

All four leading association descriptors showed progressively higher values across GMFCS levels I–III (Figure 3; Table 3). Spatial entropy showed the strongest rank association, while the two regional CCI measures and activation duty cycle showed similarly consistent monotone gradients.

In the participant-level QC analysis, retained cycle count and removed-cycle fraction were not associated with GMFCS, whereas the cycle-duration coefficient of variation showed a modest positive association. None of the 12 direct leading-feature–QC correlations survived FDR correction. After simultaneous adjustment for the three measured QC quantities, all four leading feature–GMFCS associations remained positive (partial ρ=0.656–0.825; all FDR $q<2.1\times 10^{-5}$; Supplementary Section S10).

### 3.2. Supporting Within-Cohort Multivariate Discrimination

The design-locked 16-feature Extra-Trees model yielded QWK 0.837±0.046 across 50 repeated nested-CV evaluations (Table 4). The participant-level modal OOF prediction was correct for 32/38 children (84.2%), and no I↔III error occurred.

**Figure 4:**
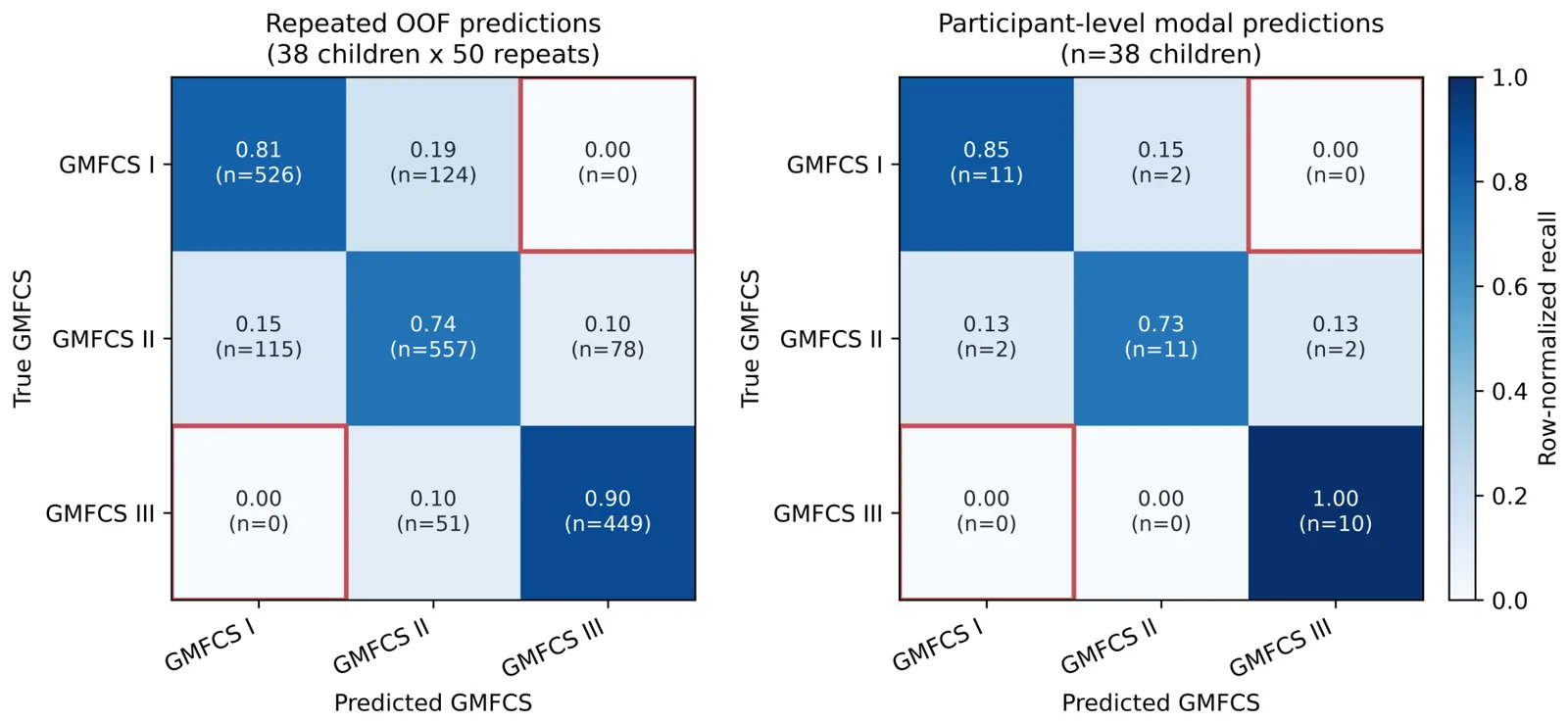
Repeated out-of-fold (OOF) predictions for the 16-feature Extra-Trees model. The left panel aggregates predictions over 50 repeats, and the right panel shows one modal prediction per participant (n=38), derived from the same repeated cross-validation predictions. Both panels are row-normalized to recall; red boxes mark the I↔III cells. GMFCS, Gross Motor Function Classification System.

Figure 4 summarizes the repeated OOF predictions; its pooled cells comprise 1900 repeated predictions from 38 children and are not independent observations. Modal prediction was the most frequent of the 50 OOF predictions for each child. Conditional on the fitted repeated-CV predictions, the subject-clustered bootstrap gave QWK 0.838 (95% confidence interval (CI) 0.718–0.920). Further participant-level stability and bootstrap details are reported in Supplementary Section S8.

### 3.3. Outer-Fold-Contained Four-Feature Selection Sensitivity

When correlation reduction, representative selection, and ranking were refitted using only the outer-training participants, the unweighted four-feature procedure yielded QWK 0.849±0.029, MAE 0.184±0.033, accuracy 0.816±0.033, and macro-$F_{1}$ 0.822±0.032 (Table 5). Precision was 0.829, 0.784, and 0.854 for GMFCS I, II, and III, respectively; recall was 0.842, 0.745, and 0.890. The conditional participant-clustered QWK interval was 0.730–0.928. In the separately seeded balanced-weighting sensitivity analysis, mean QWK was 0.871±0.025; precision was 0.839, 0.845, and 0.834, and recall was 0.886, 0.727, and 0.942 for GMFCS I, II, and III, respectively.

Every outer fold yielded 8–12 available representatives and therefore retained exactly four features. Feature identities varied: activation duty cycle, distal CCI, spatial entropy, and proximal CCI were selected in 94.4%, 91.2%, 79.6%, and 72.8% of folds, respectively. Complete metrics, per-class results, and selection frequencies are reported in Supplementary Section S15a.

The named fixed core-four candidate yielded an apparent post-selection QWK of 0.889±0.021. Because the four descriptors were selected using the complete development cohort, the QWK estimate and the associated fixed-feature permutation result are reported as descriptive post-selection results rather than evidence of predictive validity (Supplementary Section S15b).

### 3.4. Model Comparison and Balanced Sensitivity

Among the five design-locked 16-pool classifiers, Extra-Trees had the highest QWK point estimate. In the separately seeded balanced-weighting sensitivity analyses, mean QWK and GMFCS-I/III recall were higher, whereas GMFCS-II recall was lower, in both the 16-pool and fold-contained four-feature analyses. Model-family differences were not formally tested. The four-feature rows summarize a post hoc sensitivity analysis of the fold-contained selection procedure. Seven additional post hoc benchmark models are reported in Supplementary Table S7.

### 3.5. Model Size and Selection Stability

After restriction to feature counts supported within each training fold, the one-standard-error rule retained no more than five features in 84.8% of outer folds. The four leading association descriptors were also the four most frequently selected features across the 250 outer folds, with frequencies ranging from 62.8% to 94.4%; these frequencies describe selection stability within the repeated-CV analysis. EnvDuty75_mean and EnvSpectralEntropy_mean were never selected. Selection frequencies for the separate fold-contained four-feature procedure are reported in Supplementary Section S15a.

### 3.6. Open-Workbook Directional Comparison and Cross-Cohort Transportability

#### 3.6.1. Directional Comparison Under Approximate Harmonization

**Figure 5:**
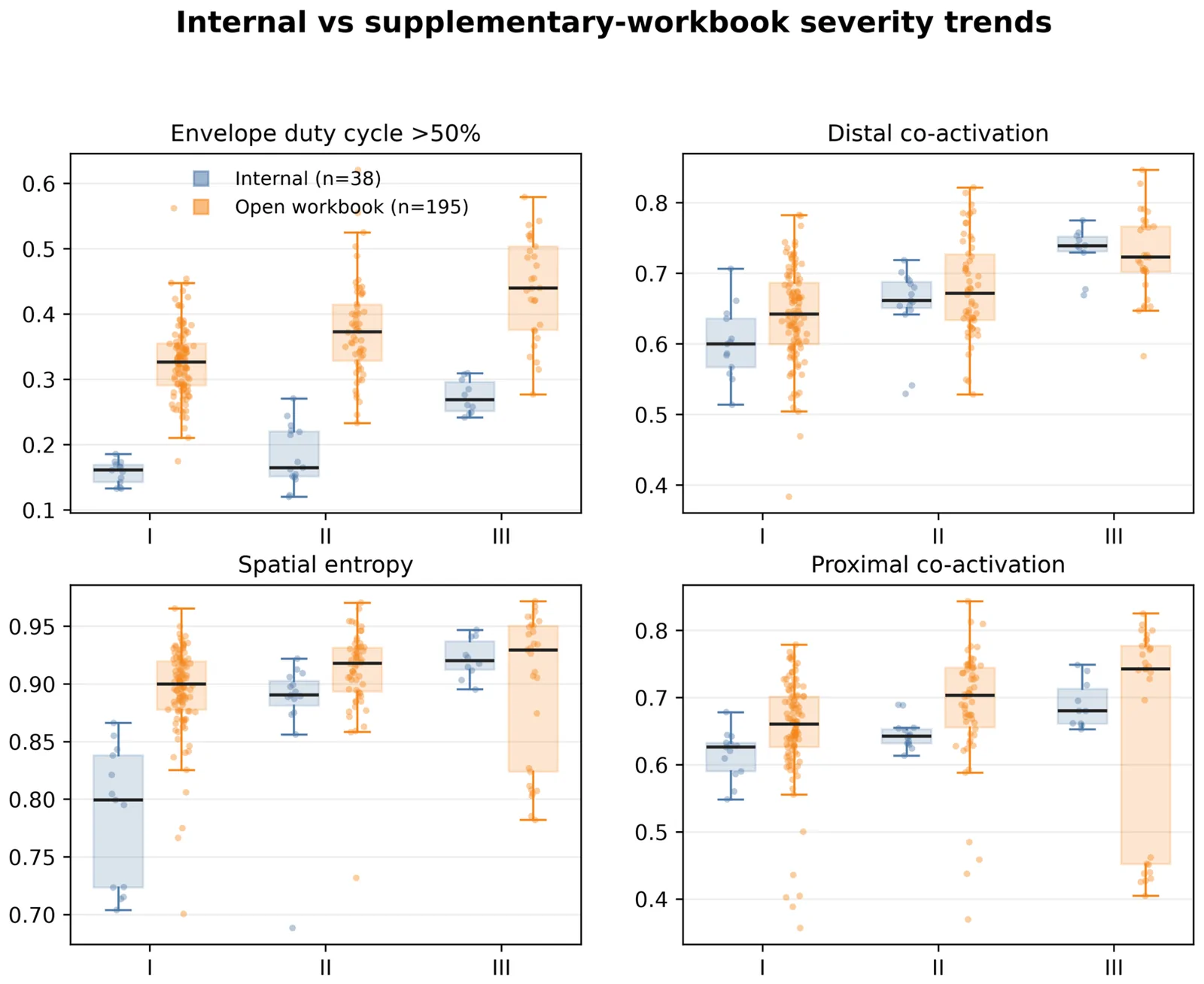
Internal (n=38) and open supplementary-workbook (n=195) GMFCS distributions for harmonized counterparts of the leading features. Points denote participants; boxes show the interquartile range and median. The comparison concerns direction across levels; open-workbook proximal and spatial descriptors include approximate muscle mappings. GMFCS, Gross Motor Function Classification System.

The open-workbook directional comparison included 195 unique participant IDs (GMFCS I/II/III =108/58/29). Duty cycle and distal peak-normalized CCI retained monotone positive associations with GMFCS (ρ=0.482 and 0.393, respectively), whereas proximal CCI and spatial entropy showed attenuated and non-monotonic associations (Figure 5). Association signs agreed between cohorts for 13/16 harmonized features. The complete paired coefficients and concordance indicators are provided in Supplementary Table S6. Excluding the seven true-equinus participants left all 16 directions unchanged and changed no coefficient by more than 0.049 (Supplementary Section S12).

#### 3.6.2. Fixed-Model Transportability Stress Tests

Direct transfer of the fixed all-16 internal classifier to the 195 open-workbook participants yielded QWK 0.210 (Table 6). The classifier predicted only one participant as GMFCS I although 108 participants were clinically classified as GMFCS I, consistent with substantial mismatch between the open-workbook feature space and the learned internal-cohort decision rules. The n=188 sensitivity result was similar (Supplementary Section S12).

The core-four candidate transported even less well (QWK 0.077); 63 of 108 open-workbook GMFCS-I participants were assigned to GMFCS III. Thus, neither the all-16 nor the core-four decision thresholds transported across acquisition and feature-generation settings.

#### 3.6.3. Post Hoc In-Domain and Domain-Shift Diagnostics

Post hoc in-domain and domain-shift analyses evaluated plausible contributors to poor direct transfer.

When retrained and evaluated within the open workbook, the all-16 and core-four specifications yielded QWK 0.559±0.031 and 0.532±0.032, respectively (Table 6). These values describe within-workbook performance after refitting; balanced-weight results are reported in Supplementary Section S16.

Cross-cohort feature-distribution shift was substantial: 14/16 features differed after FDR correction, and 11/16 had Kolmogorov–Smirnov D≥0.50. The area under the receiver operating characteristic curve (ROC-AUC) for the domain classifier was 1.000 with all 16 features and remained 0.976–0.977 with only the core four. Several feature–GMFCS associations were attenuated, most prominently for spatial entropy (ρ=0.852 internally and 0.216 in the open workbook); additional diagnostics are reported in Supplementary Section S16.

Using the complete open-workbook marginal distribution, median/IQR alignment increased all-16 direct-transfer QWK from 0.210 to 0.426, and marginal quantile alignment increased it to 0.436. The latter reduced the gap between raw transfer and unweighted open-workbook in-domain performance by approximately 65%, although a residual gap remained. These post hoc analyses served as distribution-alignment diagnostics; additional results are provided in Supplementary Section S16.

## 4. Sensitivity Analyses

### 4.1. Label Permutation

In the separate one-repeat adaptive-*k* label-permutation analysis, the matched observed QWK was 0.754. Across 1000 complete label permutations, the null mean was 0.0036 (SD 0.1847; range −0.5363 to 0.5334), and no null value reached the observed statistic. The empirical *p* value for this matched one-repeat statistic was (0+1)/(1000+1)=0.000999; adaptive *k* selection and all training-fold preprocessing were rerun for every permutation.

### 4.2. Balanced Operating Point

In the separately seeded balanced Extra-Trees sensitivity analysis, mean QWK was 0.869±0.032; GMFCS-I/III recall was 0.892/0.942, and GMFCS-II recall was 0.715, compared descriptively with 0.741 for the unweighted operating point. The prespecified unweighted analysis remained primary.

### 4.3. Cadence-Related Sensitivity

Cadence was lower at higher GMFCS levels, from 132.8±12.8 steps/min in GMFCS I to 115.7±11.5 steps/min in GMFCS III (ρ=−0.501, p=0.00137). After adjustment for cadence, all four leading associations remained positive (partial ρ=0.630–0.803; all FDR $q\leq 2.95\times 10^{-5}$; Supplementary Section S10a). Potential residual confounding by walking speed and step/stride length could not be assessed because these quantities were not measured.

### 4.4. Diplegia-Only Sensitivity

After excluding the five children with hemiplegia, the diplegia-only subset contained 33 children (GMFCS I/II/III =11/14/8). All four core features retained monotone I < II < III gradients. Spearman associations were ρ=0.700 for duty cycle, 0.713 for distal CCI, 0.826 for spatial entropy, and 0.699 for proximal CCI; all four remained significant after FDR correction ($q\leq 6.07\times 10^{-6}$). FDR-adjusted Jonckheere–Terpstra tests were also significant ($q\leq 3.59\times 10^{-5}$), and I-versus-III Cliff’s δ ranged from −1.000 to −0.909.

### 4.5. Feature-Definition Sensitivity

All 84 feature–variant evaluations (four core features × 21 processing variants) retained positive Spearman correlations with GMFCS level. Across features and variants, ρ ranged from 0.446 to 0.874, every FDR-adjusted Jonckheere–Terpstra test remained significant (q≤0.00942), and the weakest absolute GMFCS I-versus-III Cliff’s δ was 0.754. Complete feature-specific results are provided in Supplementary Table S5. All 38 recordings had at least 23 QC-retained cycles before endpoint exclusion. After the first and last retained cycles were removed, the median number of cycles analyzed per recording was 39 (range 21–59), and all four association directions were preserved: Spearman ρ=0.676–0.852, FDR-adjusted Jonckheere–Terpstra $q\leq 2.54\times 10^{-5}$, and GMFCS I-versus-III Cliff’s δ=−1.000 to −0.938 (Supplementary Section S10b and Table S10).

### 4.6. Exploratory NMF Convergence and Cycle-Sampling Stability

All NMF fits converged under the specified settings. Across candidate cycles, tVAF1 showed moderate repeatability, while the discrete synergies_95VAF measure was more sensitive to cycle selection. Full convergence and cycle-sampling results are reported in Supplementary Section S13.

## 5. Discussion

The principal finding was a small set of physiologically interpretable sEMG descriptors associated with GMFCS level: longer activation duty cycle, stronger peak-normalized distal and proximal co-activation, and a more even inter-muscle activation distribution. Because walking speed and stride length were not measured, these four gradients are interpreted in the context of self-selected walking rather than as speed-independent effects. These gradients were preserved across processing variants and after exclusion of the first and last retained cycles, adjustment for gait-cycle QC quantities and the cadence proxy, and restriction to children with diplegia. Harmonized counterparts in the open supplementary workbook provided the clearest directional support for duty cycle and distal CCI, whereas direct classifier transfer was poor.

Activation duty cycle offered the most direct temporal interpretation. Higher GMFCS level was associated with a larger fraction of the gait cycle spent above 50% of the muscle-specific 95th-percentile walking-envelope reference, consistent with a broader temporal activation profile after within-muscle normalization. Prior work examined synergy activation duration and co-activation in typically developing children and children with CP at GMFCS levels I and II, while also reporting GMFCS-related differences in synergy structure; the present duty-cycle definition and participant-level aggregation are distinct [22]. More broadly, multi-muscle sEMG has been proposed as a means of assessing neural-control impairments that are not captured by isolated clinical observations [2].

Peak-normalized regional CCI provided complementary information about coordination. Distal CCI showed high selection stability and directional consistency of its harmonized counterpart, while proximal CCI was less dominant but remained associated with higher GMFCS level after cadence-proxy adjustment. Prior CP studies and reviews have likewise treated co-activation as a relevant dimension of impaired motor control, but they vary in muscle pairs, normalization, task, and mathematical definition [22,27,28]. Averaging within-child normalized envelopes across several regional muscle pairs was intended to reduce sensitivity to common gain and isolated-site variation, although volume conduction, activation shape, and effort can still affect apparent co-activation.

Spatial entropy provided a third descriptive axis. At higher GMFCS levels, activation was distributed more evenly across the recorded muscles. Entropy-based gait classification has also been explored in children with CP using kinematic and kinetic time series [14]; however, those signal domains and entropy definitions differ from the inter-muscle spatial entropy used here, so the studies are conceptually related rather than directly comparable. Multichannel sEMG complexity in CP has also been evaluated using multivariate multiscale entropy [39]; that temporal multiscale construct is likewise distinct from the instantaneous across-muscle distribution used here. Spatial entropy was the strongest internal univariate association; its harmonized counterpart was less consistent because it depended on approximate muscle mapping, showed non-monotone level means, and had a substantially attenuated rank association.

The design-locked 16-feature analysis showed within-cohort ordinal information (QWK 0.837±0.046). When all outcome-dependent reduction and selection were repeated within each outer-training fold and exactly four features were retained, QWK was 0.849±0.029; feature identities were allowed to vary. This is an estimate of the fold-contained selection procedure, not an unbiased validation of the named core-four set. The latter’s higher apparent QWK (0.889±0.021) followed full-development-cohort selection and therefore represents a descriptive post-selection estimate rather than predictive evidence.

Duty cycle and distal CCI retained the clearest directional concordance in the open workbook, whereas proximal CCI and spatial entropy were attenuated. Direct transfer of both internal classifiers was poor despite moderate in-domain performance after refitting (all-16 QWK 0.559±0.031; core-four QWK 0.532±0.032). The source study used a different acquisition and analysis setting [23], and EMG processing choices can materially affect derived synergy outputs [26]. The marked cohort separability, feature-distribution differences, and partial recovery after marginal alignment are consistent with measurement-domain shift as one contributor to limited transportability. Thus, the cross-cohort results support selected feature directions more strongly than fitted decision thresholds; this cross-cohort comparison was a transportability stress test rather than formal external validation, and approximate harmonization does not establish clinical predictive utility.

The 16-feature analysis used a prespecified design lock, training-confined across-participant preprocessing and selection, admissible within-fold feature counts, feature-definition sensitivity analyses, QC and topography checks, and label permutation. Nevertheless, all 50 repetitions reused the same 38 children: the effective sample size remained 38, and the repeat-level SD reflects sensitivity to resampling partitions rather than independent-sample precision or generalizability [40]. This limitation is especially important for GMFCS III (n=10); the conditional 16-pool and fold-contained four-feature QWK intervals were 0.718–0.920 and 0.730–0.928. Independent prospective validation is required before individual-level or clinical use.

Other limitations include the cross-sectional design, absence of a healthy control group, absence of test–retest recordings, lack of reference-system validation for the accelerometry-derived gait-cycle boundaries, and unilateral recording in children with diplegia. Although the recorded limb was the side judged by the examining clinician to have greater clinical involvement, a unilateral recording cannot fully characterize bilateral involvement or within-participant asymmetry. The endpoint analysis evaluated sensitivity to recording boundaries but did not identify or exclude start-up, deceleration, or turning cycles. This study therefore characterizes GMFCS-related variation within ambulatory CP; comparison cohorts are needed to assess CP specificity. The associations were observed during self-selected walking and remained positive after cadence adjustment; direct walking-speed and step/stride-length measurements are needed to quantify speed-related effects. The single-cycle NMF descriptors remained sampling-dependent and were therefore exploratory rather than central predictive evidence. Raw open-workbook sEMG was unavailable, and the workbook used different preprocessing, approximate muscle substitutions, and table-derived NMF quantities; direct transfer constituted a transportability stress test under approximate harmonization. Distribution alignment used the complete unlabeled open-workbook feature distribution and was exploratory. Prospective multi-center studies should include appropriate comparison groups, prospectively standardize and document the clinical side-selection criterion or acquire bilateral sEMG, harmonize acquisition and anatomy, directly measure speed, stride length, effort, and assistive-device use, validate gait-cycle boundary detection, and repeat recordings across sessions. Such validation is important because clinical use of sEMG in CP remains constrained by limited normative resources and challenges in interpretation and standardization [2].

## 6. Conclusions

In 38 ambulatory children with spastic cerebral palsy, prolonged activation above 50% of the muscle-specific 95th-percentile walking-envelope reference, stronger peak-normalized distal and proximal co-activation, and greater inter-muscle spatial entropy were associated with higher GMFCS level. Repeated participant-level cross-validation supported within-cohort discrimination, while poor fixed-model transfer contrasted with the clearer directional concordance of duty cycle and distal CCI. These within-CP associations were observed during self-selected walking. Prospective studies with appropriate comparison groups, harmonized raw sEMG, anatomically matched channels, instrumented walking-speed and step/stride-length measurements, and repeated sessions are needed to assess specificity and generalizability. 

## Figures and Tables

**Table 1:** Participant characteristics by GMFCS level. Values are the mean ± standard deviation (SD) or *n* (%). Comparisons are descriptive; non-significant *p*-values should not be interpreted as evidence of covariate balance.

	GMFCS I (*n* = 13)	GMFCS II (*n* = 15)	GMFCS III (*n* = 10)	*p*
Sex				
Male, *n* (%)	7 (53.8%)	9 (60.0%)	5 (50.0%)	0.919
Female, *n* (%)	6 (46.2%)	6 (40.0%)	5 (50.0%)	
Topography				
Diplegia, *n* (%)	11 (84.6%)	14 (93.3%)	8 (80.0%)	0.597
Hemiplegia, *n* (%)	2 (15.4%)	1 (6.7%)	2 (20.0%)	
Antispasticity intervention	None	None	None	—
Age (y)	8.5 ± 2.3	7.9 ± 2.6	8.3 ± 2.1	0.795
Height (cm)	123.5 ± 16.2	125.7 ± 13.9	124.8 ± 14.7	0.927
Weight (kg)	26.5 ± 10.2	27.1 ± 9.8	26.1 ± 9.6	0.968

GMFCS, Gross Motor Function Classification System. By inclusion criterion, no participant had received botulinum toxin, oral baclofen, or selective dorsal rhizotomy. Sex and topography *p*-values are from two-sided Fisher–Freeman–Halton exact tests; continuous *p*-values are from Kruskal–Wallis tests across levels.

**Table 2:** Analytical roles and development sequence. “Design-locked” indicates that the feature pool was fixed before the reported final evaluation. Association, within-cohort discrimination, and external predictive utility are separate inferential targets.

Level	Analysis	Role	Permitted Interpretation
Primary association analysis	Feature–GMFCS associations	Direction, magnitude, and physiological interpretation of GMFCS-related sEMG differences	Feature direction, magnitude, and interpretation
Within-cohort multivariate analysis	Adaptive Extra-Trees using the 16-feature pool	Test whether the locked representation contains participant-level ordinal information	Repeated-CV discrimination in these 38 children
Four-feature selection sensitivity	Training-fold correlation reduction and ANOVA ranking, followed by fixed Extra-Trees	Estimate a fold-contained four-feature selection procedure	Internal performance of the procedure, not of one named feature set
Post hoc fixed candidate	Fixed Extra-Trees using the named four descriptors	Describe the development-set specification and assess transportability	Apparent internal description and external stress test only

**Table 3:** Primary internal-cohort results for the four leading association descriptors. Gross Motor Function Classification System (GMFCS) columns report the mean ± standard deviation (SD). Spearman and Cliff’s δ intervals are 95% participant-level bootstrap confidence intervals (CIs). The *q* values are adjusted for the false discovery rate (FDR) within the 16-feature descriptive family. Cliff’s δ contrasts GMFCS I minus III; negative values indicate higher values in GMFCS III. CCI, co-activation index; JT, Jonckheere–Terpstra.

**Feature**	**GMFCS I Mean ± SD**	**GMFCS II Mean ± SD**	**GMFCS III Mean ± SD**	**Spearman ρ (95% CI)**	**Spearman FDR *q***
Activation duty cycle	0.157±0.017	0.183±0.046	0.274±0.026	0.683 (0.549–0.811)	$6.22\times 10^{-6}$
Distal peak-normalized CCI	0.601±0.051	0.655±0.053	0.733±0.034	0.751 (0.592–0.883)	$4.42\times 10^{-7}$
Spatial entropy	0.785±0.060	0.879±0.055	0.922±0.017	0.852 (0.751–0.927)	$1.87\times 10^{-10}$
Proximal peak-normalized CCI	0.615±0.036	0.645±0.021	0.690±0.035	0.736 (0.571–0.864)	$6.36\times 10^{-7}$
**Feature**	**JT FDR q**		**GMFCS I-minus-III Cliff’s δ (95% CI)**		
Activation duty cycle	$5.34\times 10^{-5}$		−1.000 (−1.000 to −1.000)		
Distal peak-normalized CCI	$9.54\times 10^{-6}$		−0.969 (−1.000 to −0.862)		
Spatial entropy	$2.77\times 10^{-7}$		−1.000 (−1.000 to −1.000)		
Proximal peak-normalized CCI	$9.54\times 10^{-6}$		−0.938 (−1.000 to −0.769)		

**Table 4:** Design-locked 16-pool Extra-Trees performance under 50 repetitions of stratified five-fold nested cross-validation in the same 38 children. Values are the mean ± standard deviation (SD) across resampling repeats where available. MAE, mean absolute error; QWK, quadratic weighted kappa.

Metric	Result
QWK	0.837±0.046
Ordinal MAE	0.196±0.051
Accuracy	0.804±0.051
Macro-$F_{1}$	0.811±0.049
Recall I/II/III	0.805/0.741/0.898
Precision I/II/III	0.818/0.762/0.850
I→III/III→I	0/0

**Table 5:** Participant-level repeated-CV performance of the design-locked 16-pool classifiers and sensitivity analyses on 50 × 5 outer partitions. For the four-feature procedure, correlation reduction and outcome-dependent ranking were repeated in every outer-training fold. QWK is reported as mean ± standard deviation (SD) across repeats; MAE, accuracy, macro-$F_{1}$, and class-specific recall are repeat-averaged values. The same 38 children were reused across repeats. Acc., accuracy; MAE, mean absolute error; QWK, quadratic weighted kappa; RBF, radial basis function; SVM, support vector machine.

Model	QWK	MAE	Acc.	Macro-$F_{1}$	Recall I/II/III
Linear SVM	0.793±0.051	0.239	0.761	0.770	0.734/0.740/0.828
RBF SVM	0.812±0.051	0.218	0.782	0.790	0.749/0.765/0.850
Random forest	0.817±0.049	0.218	0.782	0.790	0.777/0.728/0.870
Penalized multinomial logistic regression	0.793±0.049	0.244	0.756	0.764	0.711/0.719/0.870
Extra-Trees (16-pool, prespecified support)	0.837±0.046	0.196	0.804	0.811	0.805/0.741/0.898
Balanced Extra-Trees (16-pool sensitivity)	0.869±0.032	0.165	0.835	0.838	0.892/0.715/0.942
Outer-fold-selected Extra-Trees (four-feature sensitivity)	0.849±0.029	0.184	0.816	0.822	0.842/0.745/0.890
Balanced outer-fold-selected Extra-Trees (four-feature sensitivity)	0.871±0.025	0.162	0.838	0.841	0.886/0.727/0.942

**Table 6:** Fixed-model transportability stress tests and within-workbook refitted performance for the all-16 and core-four Extra-Trees specifications. Direct transfer used classifiers fitted only on the 38 internal participants and is reported as a point estimate; refitted entries are the mean ± standard deviation (SD) across 50 repeated five-fold cross-validation (CV) evaluations. Balanced-weight results are reported in Supplementary Section S16. MAE, mean absolute error; QWK, quadratic weighted kappa.

Evaluation Setting	QWK	MAE	Accuracy	Balanced Accuracy	Macro-$F_{1}$
All-16 fixed-model transportability stress test	0.210	0.836	0.277	0.428	0.266
All-16 within-workbook refitted performance	0.559±0.031	0.400±0.019	0.636±0.016	0.546±0.020	0.543±0.023
Core-four fixed-model transportability stress test	0.077	1.128	0.215	0.366	0.203
Core-four within-workbook refitted performance	0.532±0.032	0.409±0.021	0.639±0.016	0.553±0.022	0.551±0.025

## Data Availability

The internal-cohort data presented in this study are available on request from the corresponding author and are not publicly available due to privacy and ethical restrictions. The open-workbook directional comparison reused the workbook published as Table S1 accompanying Goudriaan et al. [23], available under CC BY from Figshare (https://doi.org/10.6084/m9.figshare.13123175.v1; data citation [38]). This workbook is supplementary data for the cited article rather than a separately collected dataset.

## Supplementary Materials

The following supporting information can be downloaded at https://www.mdpi.com/article/10.3390/s26175434/s1: Supplementary.pdf: Supplementary Methods, Results, and Tables (Sections S1–S16); Table S1: Internal-cohort statistics for all 16 design-locked features. False discovery rate (FDR) correction was performed within the 16-feature descriptive family. Cliff’s δ is I–II/II–III/I–III; negative values indicate larger values in the higher-GMFCS group. Selection frequency is selection in the 250 outer folds. Bootstrap confidence intervals are reported for the four core features in PDF Table S8a and for all 16 features in Supplementary Table S8b. CCI, co-activation index; GMFCS, Gross Motor Function Classification System; JT, Jonckheere–Terpstra; KW, Kruskal–Wallis; VAF, variance accounted for. Table S2: Directional evaluation of harmonized counterparts of the 16 features in 195 unique participants from the open workbook (Table S1 accompanying Goudriaan et al.). Sample inclusion and the n=188 sensitivity analysis are described in Section S12. Several descriptors used approximate muscle mapping or table-derived NMF quantities and are not exact reproductions. Absolute values are cohort- and preprocessing-specific; inference concerns within-workbook GMFCS trends. All three *q*-value columns report Benjamini–Hochberg adjustment across the 16 features within the corresponding test family. GMFCS, Gross Motor Function Classification System; NMF, non-negative matrix factorization. Table S3: Gait-cycle boundary detection and quality-control summary by GMFCS level. Values are participant-level median [range] unless otherwise stated. GMFCS, Gross Motor Function Classification System; LG, lateral gastrocnemius; SD, standard deviation; SO, soleus; TA, tibialis anterior. Table S4: Summary of feature-definition sensitivity analyses. The largest JT *q* is the least favorable false-discovery-rate-adjusted Jonckheere–Terpstra value across variants; weakest |δ| is the smallest absolute GMFCS I-versus-III Cliff’s effect. GMFCS, Gross Motor Function Classification System; JT, Jonckheere–Terpstra. Supplementary Table S5 (machine-readable). Complete feature-definition sensitivity results for all 84 feature–variant evaluations are provided in Supplementary_Table_S5_feature_definition_sensitivity_84_evaluations.csv. Supplementary Table S6 (machine-readable). Internal- and open-workbook feature-direction concordance results are provided in Supplementary_Table_S6_internal_open_workbook_direction_concordance.csv. Table S7: Additional post hoc model-family benchmarks under the same 50×5 outer partitions used for the design-locked 16-feature analysis. These additional model-family analyses were post hoc and are reported as contextual benchmarks. QWK is reported as mean ± standard deviation across repeats; MAE, accuracy, macro-$F_{1}$, and class-specific recall are repeat-averaged values. These summaries describe resampling variability rather than independent-sample uncertainty. LDA, linear discriminant analysis; MAE, mean absolute error; QWK, quadratic weighted kappa. Table S8a: Participant-level stratified percentile-bootstrap 95% confidence intervals for the four core feature effect sizes (B=10,000; seed 20260724). Cliff’s δ uses the GMFCS I-minus-III convention; negative values indicate larger values in GMFCS III. Supplementary Table S8b reports all 16 design-locked features. CCI, co-activation index; CI, confidence interval; GMFCS, Gross Motor Function Classification System. Supplementary Table S8b (machine-readable). Bootstrap confidence intervals for effect sizes of all 16 design-locked features are provided in Supplementary_Table_S8b_effect_size_bootstrap_ci_all16.csv. Table S9a: Locked transfer and diagnostic alignment analyses in the 195-participant open-workbook sample. Alignment used the complete unlabeled open-workbook marginal distribution as a post hoc diagnostic analysis. IQR, interquartile range; MAE, mean absolute error; QWK, quadratic weighted kappa. Table S9b: Largest marginal shifts among the 16 harmonized features. *D* is the two-sample Kolmogorov–Smirnov statistic. Fourteen of 16 features remained different after Benjamini–Hochberg correction, and 11/16 had D≥0.50. KS, Kolmogorov–Smirnov. Supplementary Table S10 (machine-readable). Feature-level recording-endpoint cycle-exclusion sensitivity results are provided in Supplementary_Table_S10_recording_endpoint_cycle_sensitivity.csv. Additional supporting archives: Supplementary_RecordingEndpointCycle_Analysis.zip, “Supporting Materials for the Recording-Endpoint Cycle-Exclusion Sensitivity Analysis”; Supplementary_NMF_Stability_Analysis.zip, “NMF Convergence and Cycle-Sampling Results”; Supplementary_OuterFoldFourFeature_Analysis.zip, “Outer-Fold-Contained Four-Feature Results”; and Supplementary_FixedCoreFour_Analysis.zip, “Post-Selection Fixed Four-Feature Results”.

## Abbreviations

The following abbreviations are used in this manuscript:

ANOVA	Analysis of variance
BF	Biceps femoris
CCI	Co-activation index
CI	Confidence interval
CP	Cerebral palsy
CV	Cross-validation
EMG	Electromyography
FDR	False discovery rate
GM	Gluteus medius
GMFCS	Gross Motor Function Classification System
IIR	Infinite impulse response
IMU	Inertial measurement unit
IQR	Interquartile range
JT	Jonckheere–Terpstra
kNN	*k*-nearest neighbors
LDA	Linear discriminant analysis
LG	Lateral gastrocnemius
MAD	Median absolute deviation
MAE	Mean absolute error
MH	Medial hamstrings
ML	Machine learning
NMF	Non-negative matrix factorization
OOF	Out-of-fold
QC	Quality control
QDA	Quadratic discriminant analysis
QWK	Quadratic weighted kappa
RBF	Radial basis function
RF	Rectus femoris
ROC-AUC	Area under the receiver operating characteristic curve
SD	Standard deviation
SE	Semitendinosus
SENIAM	Surface Electromyography for the Non-Invasive Assessment of Muscles
SHA-256	256-bit Secure Hash Algorithm
sEMG	Surface electromyography
SO	Soleus
SVM	Support vector machine
TA	Tibialis anterior
TFL	Tensor fasciae latae
VAF	Variance accounted for
VL	Vastus lateralis
