# Supplementary material for: Activation Duty Cycle and Regional Muscle Co-Activation Across GMFCS Levels in Ambulatory Children with Spastic Cerebral Palsy: An Exploratory Surface EMG Study
Source: Sensors (Basel). 2026 Aug 27;26(17):5434. doi: 10.3390/s26175434 (PMC13568141; doi:10.3390/s26175434)
Supplement: Supplementary file 1 [file sensors-26-05434-s001.zip › Supplementary.pdf]

Supplementary Materials for: Activation Duty Cycle and Regional Muscle Co-Activation across GMFCS Levels in Ambulatory Children with Spastic Cerebral Palsy: An Exploratory Surface-EMG Study

Peng Huang, Zhen Zhang, Yonggang Tong, and Yingfei Sun

Abbreviations. BF, biceps femoris; CCI, co-activation index; CI, confidence interval; CV, cross-validation; EMG, electromyography; FDR, false discovery rate; GM, gluteus medius; GMFCS, Gross Motor Function Classification System; ICC, intraclass correlation coefficient; IQR, interquartile range; JT, Jonckheere–Terpstra; LG, lateral gastrocnemius; MAD, median absolute deviation; MAE, mean absolute error; MH, medial hamstrings; NMF, non-negative matrix factorization; OOF, out-of-fold; PSD, power spectral density; QC, quality control; QWK, quadratic weighted kappa; RF, rectus femoris; SD, standard deviation; SE, semitendinosus; SHA-256, 256-bit Secure Hash Algorithm; SO, soleus; SSE, sum of squared errors; TA, tibialis anterior; TFL, tensor fasciae latae; VAF, variance accounted for; VL, vastus lateralis.

Supplementary Methods and Results

S1. Design rationale and locking chronology

The 16-feature pool was fixed before the final 50-repeat evaluation based on measurement and portability considerations. It emphasized aggregate peak-normalized envelope timing, regional co-activation, spatial distribution, envelope-frequency shape, and single-cycle synergy descriptors; absolute-amplitude, raw-EMG spectral, individual-pair, and local extreme or dispersion features were excluded.

S2. Peak normalization and regional definitions

For each participant and muscle independently, all quality-controlled cycles and all 101 time-normalized envelope samples were pooled, and the envelope was divided by that muscle's 95th percentile. Task-based peak amplitude is a recognized normalization category; the 95th percentile was an operational choice intended to limit the influence of isolated extreme samples, rather than a percentile prescribed by the cited consensus source. No cohort-level amplitude reference was used. Distal muscles were SO, TA, and LG; proximal muscles were VL, RF, SE, BF, and TFL. For each within-region pair, peak-normalized samples were concatenated across cycles and CCI was calculated as $2\sum\min(E_i, E_j)/(\sum E_i + \sum E_j)$. Regional features are means over within-region pairs. In the open workbook (Table S1 accompanying Goudriaan et al.), SO, TA, VL, RF, and BF mapped directly, and LG mapped to the available gastrocnemius slot. Medial hamstrings (MH) occupied the internal SE slot as an approximate hamstring mapping; gluteus medius (GM) occupied the internal TFL slot despite not being anatomically equivalent. Descriptors involving these substitutions are harmonized counterparts rather than exact reproductions.

S2a. Additional gait-cycle boundary-detection details

Positive and negative peak polarities were evaluated, and the polarity yielding more valid 0.70–1.60 s inter-peak intervals was retained; total peak count resolved any tie. The TA/LG/SO ankle-region composite was available for all 38 recordings, so neither the standalone ACC10 channel nor fixed 1-s segmentation was required.

S3. Envelope timing, distribution, and frequency-shape descriptors

Duty-cycle descriptors are the fractions of peak-normalized samples exceeding 0.25, 0.50, and 0.75, calculated by muscle and averaged over eight muscles. A burst is a contiguous within-cycle run above 0.50,

with no minimum-duration filter; EnvBurstDuration_mean is the mean run length divided by 101 across all detected bursts, muscles, and cycles, or 0 when no burst is detected. Peak timing is the normalized within-cycle argmax location, and its sample SD is calculated across muscles and cycles. For each muscle, peak-normalized cycles are concatenated and demeaned before Welch estimation at a nominal 100 samples per cycle, using a periodic Hann window, nperseg= $\min(N, 256)$, 50% overlap, constant detrending, nfft= nperseg, and no frequency interpolation. Envelope centroid is the PSD-weighted centroid within 1–12 cycles^{-1}; norm denotes normalized-envelope input and not division by Nyquist. The high/low ratio compares power at 6–12 versus 1–3 cycles^{-1}, and envelope spectral entropy is calculated within 1–12 cycles^{-1}. Spatial entropy is $-\sum_m p_m \log(p_m + 10^{-12})/\log 8$ at each concatenated time sample; zero-total-envelope samples are omitted, and the result is missing only when all samples have zero total envelope. For EnvIntermuscleCorr_mean, the normalized cycles are concatenated within muscle, Pearson r is calculated for each of the 28 muscle pairs, and finite coefficients are averaged without Fisher-z transformation. The distal/proximal envelope ratio provides the remaining distribution summary.

S4. Single-cycle NMF descriptors

One gait cycle per child was selected using a pseudorandom generator with a deterministic participant-specific seed and was peak-normalized by muscle. NMF models with $k = 1$–4 used 20 random initializations per k, multiplicative updates, Frobenius loss, a maximum of 1500 iterations, and tolerance 10^{-4}. Restart seeds were deterministically offset from the participant seed, and the solution with the smallest reconstruction sum of squared errors was retained. Uncentered VAF was used. SingleCyclePeakNorm_tVAF1 is the one-synergy total VAF. SingleCyclePeakNorm_synergies_95VAF is the smallest k attaining VAF ≥ 0.95, coded as 5 if no value from 1 to 4 reached the threshold. Convergence and cycle-sampling sensitivity were evaluated under the same NMF settings across all quality-controlled cycles. For the supplementary-workbook directional comparison, these variables were obtained from the deposited VAF tables and treated as harmonized counterparts. Neither NMF descriptor belonged to the four leading association descriptors; because both locked values also depended on one selected cycle, the NMF analyses were exploratory and not central predictive evidence.

S5. Label permutation and classifier-transfer sensitivity

For label permutation, the observed and null statistics both used one stratified 5-fold outer repeat with adaptive one-standard-error k selection and restriction to feature counts supported within the training fold. Each of 1000 permutations repeated training-fold median imputation, correlation clustering, representative selection and ranking, inner-CV adaptive k selection, and 500-tree Extra-Trees fitting. The matched observed QWK was 0.754; null mean±SD was 0.0036 ± 0.1847 (range -0.5363 to 0.5334), with 0/1000 values at least as large as observed, giving $p = (0+1)/(1000+1) = 0.000999$. This test remained a one-repeat statistic rather than a permutation test of the 50-repeat mean QWK. For the classifier-transfer sensitivity analysis, the prespecified 500-tree, leaf-size-2 model fitted on the internal cohort was applied unchanged to the supplementary-workbook cohort after median imputation fitted on internal data only, producing QWK 0.210 in $n = 195$. Details of the $n = 195$ and $n = 188$ samples and the exclusion sensitivity analysis are provided in Section S12; domain-shift diagnostics are provided in Section S16. The open-workbook sample contained no duplicate participant IDs; each participant contributed one record for the concatenated included gait cycles rather than separate trial-, cycle-, limb-, or session-level observations. Direct muscle mapping was available for SO, TA, VL, RF, and BF, with LG aligned to the available gastrocnemius slot; MH-to-SE and especially GM-to-TFL were approximate or anatomically non-equivalent substitutions, and the NMF quantities were table-derived.

S6. Software and reproducibility

The analysis used Python, NumPy, pandas, SciPy, scikit-learn, and statsmodels. Random-state settings were recorded as follows; repeat and fold indices were zero-based.

Analysis component	Random-state specification
Design-locked 16-feature repeated CV	For the unweighted analysis, outer-fold shuffling and the fitted classifier used the repeat index (0–49). Within outer fold f of repeat r, inner three-fold shuffling used $101r + f$. The balanced-weight sensitivity added an offset of 10,000 to these analysis seeds.
Outer-fold-contained four-feature sensitivity	Stratified outer-fold shuffling and fitted Extra-Trees used repeat indices 0–49 for the unweighted analysis and 10,000–10,049 for the balanced sensitivity. Correlation reduction, representative selection, and ANOVA ranking were refitted inside each outer-training fold. Every fold yielded at least four representatives, and the four highest-ranked representatives were retained.
Post hoc fixed core-four repeated CV	Outer-fold shuffling and the fitted classifier used the repeat index (0–49); the balanced-weight sensitivity used 10,000 plus the repeat index. The open-workbook in-domain repeated-CV analyses used the same respective rules. No inner CV was used because the feature set and model hyperparameters were fixed; this did not nest the prior full-cohort selection of the named features.
Design-locked label permutation	Label permutations used seed 20260719. The matched one-repeat outer split used seed 4242; its inner-fold and estimator seeds followed the same deterministic fold rules as the design-locked analysis.
Core-four label permutation	Label permutations used seed 20260724. The matched outer split used seed 0. Observed fold-specific estimator seeds were the fold indices; null estimator seeds were 1000 plus the permutation index plus the fold index.
Subject-grouped bootstrap	The design-locked 16-feature and core-four conditional bootstraps used seeds 20260711 and 20260724, respectively.
Feature-effect bootstrap	Spearman ρ and GMFCS I-minus-III Cliff's δ intervals used 10,000 participant-level stratified resamples with seed 20260724.
Single-cycle NMF	Each participant seed was the unsigned integer represented by the first four bytes of the SHA-256 digest of the participant identifier. The selected cycle used that participant seed; restart seed was participant seed $+ k + 1009\times$ restart index.
Fixed-model transfer and within-workbook refits	Seed 999.

S7. Analysis unit, missingness, and gait-cycle quality control

The analysis matrix contained one participant-level row for each of 38 distinct children. Signal conditioning, within-child normalization, gait-cycle aggregation, and feature extraction produced this matrix before cross-validation; these operations did not combine information across children or use GMFCS labels. Within each outer fold, the training and test sets were participant-disjoint, and all cycles, muscles, and time points from a given child remained within the same partition. Every child appeared in one test fold per repeat. The 50 repetitions reused the same 38 children and did not increase the effective sample size.

S8. Effect-size and conditional prediction bootstrap intervals

For feature-level effect sizes, 10,000 participant-level percentile-bootstrap resamples were drawn independently within GMFCS I, II, and III, thereby retaining the observed group sizes of 13, 15, and 10. Spearman ρ was recalculated after combining the three resampled groups, and Cliff's δ was recalculated for the GMFCS I-minus-III contrast. These intervals are conditional on the observed 38-child association dataset. Boundary intervals of -1.000 to -1.000 occurred when every observed and resampled GMFCS-I value was below every GMFCS-III value. PDF Table S8a reports the four core features; Supplementary

Table S8b reports all 16 design-locked features.

The subject-clustered bootstrap resampled children from the existing repeated OOF prediction table and carried all 50 predictions for each sampled child together; the resulting intervals are conditional on the fitted repeated-CV predictions. Across the 50 repeats, QWK had a median of 0.850, an interquartile range of 0.808–0.869, and a range of 0.726–0.916. Modal prediction was the most frequent of the 50 OOF predictions; when frequencies were tied, the lower coded level was assigned, although no ties occurred. Across the 38 children, modal prediction was correct for 32 (84.2%), predictions were identical across all 50 repeats for 21 (55.3%), all prediction distributions remained within a single adjacent-level span, and no child was predicted as both GMFCS I and GMFCS III. Conditional subject-clustered bootstrap estimates were QWK 0.838 (95% CI 0.718–0.920), accuracy 0.804 (0.701–0.902), and macro-F_1 0.812 (0.700–0.903); bootstrap recall intervals were 0.618–0.953, 0.536–0.930, and 0.789–0.987 for GMFCS I, II, and III, respectively.

S9. Feature-definition sensitivity analyses

The four core features were recomputed under 21 processing variants: alternative peak-normalization percentiles (90th, 95th, and 99th), envelope smoothing windows (5, 10, 20, and 50 ms), gait-cycle duration MAD thresholds (3.0, 3.5, and 4.0), cycle-duration gates (0.70–1.60 and 0.60–1.80 s), leave-one-muscle-out variants for all eight muscles, and removal of the most duration-extreme cycle in each recording. Thus the analysis comprised four core features $\times$ 21 variants = 84 feature–variant evaluations. Jonckheere–Terpstra p values were FDR corrected across the four core features within each sensitivity variant. All 84 evaluations retained Spearman $\rho > 0$ and GMFCS I-versus-III Cliff's $\delta < 0$. These within-cohort evaluations assessed the stability of the four feature gradients across the specified processing choices. Complete results are provided in Supplementary Table S5.

S10. QC-related sensitivity analysis

Participant-level QC values were merged one-to-one with the four core features by participant ID. The fraction of removed cycles was calculated as $1 - n_{\text{kept}}/n_{\text{initial}}$. The retained cycle count was 44.38 ± 10.53, 36.60 ± 8.26, and 42.80 ± 8.12 for GMFCS I, II, and III; the cycle-duration coefficient of variation was $11.50 \pm 4.73\%$, $13.66 \pm 6.65\%$, and $16.97 \pm 4.62\%$; the fraction of removed cycles was $5.35 \pm 5.89\%$, $2.69 \pm 3.19\%$, and $4.08 \pm 3.94\%$. Retained count and removed fraction were not associated with GMFCS (Spearman $\rho = -0.078$ and -0.059; both FDR $q = 0.724$), while the duration coefficient of variation showed a modest positive association ($\rho = 0.387$, $q = 0.0495$). Across the 12 core-feature–QC correlations, none survived FDR correction (minimum $q = 0.191$). Simultaneous partial-rank adjustment for all three QC quantities retained positive feature–GMFCS associations for duty cycle, distal CCI, spatial entropy, and proximal CCI (partial $\rho = 0.656$, 0.730, 0.825, and 0.713; respective FDR $q = 1.88 \times 10^{-5}$, 1.29×10^{-6}, 4.43×10^{-9}, and 2.06×10^{-6}).

S10a. Cadence-related sensitivity analysis

Cadence was 132.8 ± 12.8, 126.0 ± 11.0, and 115.7 ± 11.5 steps/min for GMFCS I, II, and III, respectively (Spearman $\rho = -0.501$, $p = 0.00137$; Kruskal–Wallis $p = 0.00814$). After controlling for cadence, associations with GMFCS remained positive for duty cycle (partial $\rho = 0.630$, FDR $q = 2.95 \times 10^{-5}$), distal CCI ($\rho = 0.674$, $q = 6.36 \times 10^{-6}$), spatial entropy ($\rho = 0.803$, $q = 8.68 \times 10^{-9}$), and proximal CCI ($\rho = 0.682$, $q = 6.36 \times 10^{-6}$). Cadence was treated as a limited proxy because walking speed also depends on step length; direct walking-speed and step/stride-length measurements are needed to quantify speed-related effects.

S10b. Recording-endpoint cycle-exclusion sensitivity analysis

The walking protocol did not distinguish steady-state walking from start-up, deceleration, or turning segments. We therefore removed the first and last chronologically ordered QC-retained cycles from each recording and recomputed the four leading descriptors using the same 95th-percentile normalization and association statistics. All recordings had at least 23 retained cycles before exclusion (median 41; range 23–61); the median after exclusion was 39 (range 21–59). All four feature–GMFCS directions were preserved: Spearman ρ ranged from 0.676 to 0.852, the largest FDR-adjusted Jonckheere–Terpstra q was 2.54×10^{-5}, and GMFCS I-minus-III Cliff's δ ranged from -1.000 to -0.938. Feature-level results are provided in Supplementary Table S10. This analysis evaluated sensitivity to recording boundaries, but it did not identify or exclude start-up, deceleration, or turning cycles.

S11. Diplegia-only sensitivity analysis

The five children with hemiplegia were excluded, leaving 33 children with diplegia (GMFCS I/II/III = 11/14/8). All four core features retained monotone I<II<III gradients. For EnvDuty50_mean, level means were 0.153 ± 0.016, 0.186 ± 0.047, and 0.278 ± 0.027; Spearman $\rho = 0.700$ (FDR $q = 6.07 \times 10^{-6}$), Jonckheere–Terpstra $z = 4.133$ ($q = 3.59 \times 10^{-5}$), and I-versus-III Cliff's $\delta = -1.000$. Corresponding results were 0.597 ± 0.054, 0.654 ± 0.055, and 0.727 ± 0.036 for distal CCI ($\rho = 0.713$, $q = 6.07 \times 10^{-6}$; JT $z = 4.266$, $q = 3.08 \times 10^{-5}$; $\delta = -0.955$); 0.779 ± 0.064, 0.879 ± 0.057, and 0.920 ± 0.018 for spatial entropy ($\rho = 0.826$, $q = 1.33 \times 10^{-8}$; JT $z = 5.032$, $q = 1.94 \times 10^{-6}$; $\delta = -1.000$); and 0.617 ± 0.038, 0.642 ± 0.018, and 0.687 ± 0.038 for proximal CCI ($\rho = 0.699$, $q = 6.07 \times 10^{-6}$; JT $z = 4.233$, $q = 3.08 \times 10^{-5}$; $\delta = -0.909$). Negative Cliff's δ indicates higher values in GMFCS III under the I-minus-III convention. The four core gradients persisted in the diplegia-only subset.

S12. True-equinus exclusion sensitivity in the supplementary workbook

The seven supplementary-workbook participants not included in the source article's 188-child gait-pattern comparison comprised the entire true-equinus group (GMFCS I/II/III = 2/3/2). The source article excluded this group from gait-pattern comparisons because it did not meet the prespecified group-size requirement; all seven participants had complete sEMG variables for the present GMFCS analysis. Because the present analysis concerned GMFCS associations, all 195 eligible participants were retained in the primary supplementary-workbook analysis. Repeating the analysis after excluding these seven participants left the Spearman direction unchanged for all 16 features; the maximum absolute change in ρ was 0.049. Distal CCI changed from $\rho = 0.393$ to 0.388, EnvDuty50_mean from 0.482 to 0.531, and SingleCyclePeakNorm_tVAF1 from 0.586 to 0.583. Fourteen of 16 FDR significance classifications were unchanged. The two changes were confined to non-core boundary features: EnvBurstDuration_mean ($q = 0.098$ to 0.0498) and DistalProximalEnvelopeRatio ($q = 0.0559$ to 0.048). Fixed-model transfer using the locked leaf-size-2 configuration also remained poor and nearly unchanged (QWK 0.210 to 0.208; accuracy 0.277 to 0.271; macro-F_1 0.266 to 0.262). The feature-direction and transfer conclusions were unchanged after exclusion of the true-equinus group.

S13. NMF convergence and cycle-sampling stability

The NMF sensitivity analysis applied the original single-cycle peak-normalized settings to every available QC-passed cycle. There were 1554 candidate cycles across 38 children (minimum/median/maximum 23/41/61 cycles). All fits converged within the 1500-iteration limit for $k = 1$–4, and recomputation of the originally selected cycles reproduced both locked descriptors.

Cycle-level stability was evaluated across all candidate cycles. The median within-child SD of tVAF1 was 0.0304 (maximum 0.0699). In 1000 balanced resamples that selected 23 cycles without replacement from every child, the median one-way random-effects ICC(1,1) was 0.602 (2.5th–97.5th percentile 0.575–0.631). The originally selected-cycle tVAF1 association with GMFCS was $\rho = 0.488$ ($p = 0.00188$); using each child's mean across all available cycles strengthened the association to $\rho = 0.633$ ($p = 1.99 \times 10^{-5}$). The selected discrete synergies_95VAF value equaled the within-child modal cycle value in 30/38 children, and the median fraction of cycles at the modal value was 66.4%. The descriptors showed moderate single-cycle sampling dependence, particularly for the discretized synergy count. These findings indicate that

the NMF descriptors are exploratory and should not be treated as central predictive evidence.

S14. Logistic-regression convergence and balanced weighting

The penalized multinomial logistic-regression comparator used training-fold standardization, the SAGA solver, elastic-net regularization (l_1 ratio 0.5, $C = 0.5$), and a maximum of 5000 iterations. Convergence was evaluated for $k \in \{3, 5, 8, 10\}$, restricted to feature counts supported within each training fold; all fitted models converged under these settings. No oversampling, undersampling, or synthetic sampling was used. The balanced Extra-Trees sensitivity analyses used the same Extra-Trees parameters with class_weight='balanced'. Within each training partition, class j was assigned $w_j = n_{\text{train}}/(3n_j)$ using only that partition's class counts.

S15a. Outer-fold-contained four-feature selection sensitivity

Within each of 50 repetitions of participant-level stratified five-fold CV, median imputation, absolute-Spearman correlation clustering at 0.8, cluster-representative selection, and ANOVA-F ranking were fitted using only the outer-training participants. Every outer-training fold yielded at least four representatives; the four highest-ranked representatives were supplied to the fixed 500-tree Extra-Trees classifier, and feature identities could vary across folds. The untouched outer-test fold contributed to none of these steps.

For the unweighted procedure, repeat-level mean±SD was QWK 0.8485 ± 0.0291, MAE 0.1837 ± 0.0326, accuracy 0.8163 ± 0.0326, balanced accuracy 0.8256 ± 0.0352, and macro-F_1 0.8217 ± 0.0324. Precision was 0.8290, 0.7842, and 0.8538 for GMFCS I, II, and III, respectively; recall was 0.8415, 0.7453, and 0.8900; and F_1 was 0.8346, 0.7621, and 0.8685. The conditional participant-clustered bootstrap QWK interval was 0.7303–0.9284.

With training-fold balanced class weighting, QWK was 0.8705 ± 0.0250, MAE 0.1621 ± 0.0298, accuracy 0.8379 ± 0.0298, balanced accuracy 0.8516 ± 0.0307, and macro-F_1 0.8412 ± 0.0291. Class-specific precision was 0.8385 / 0.8452 / 0.8343; recall was 0.8862 / 0.7267 / 0.9420; and F_1 was 0.8606 / 0.7799 / 0.8832. The conditional QWK interval was 0.7606–0.9436. Because the balanced analysis used a separate prespecified seed range, it is a sensitivity operating point rather than a same-fold paired comparison.

In the unweighted analysis, selection counts were 236/250 for EnvDuty50_mean, 228/250 for CCI_peaknorm_distal_mean, 199/250 for SpatialEntropy_mean, 182/250 for CCI_peaknorm_proximal_mean, 87/250 for EnvCentroid_norm_mean, 54/250 for EnvHL_ratio_high_over_low, and 14/250 for EnvDuty25_mean; all remaining features were selected in 0/250 folds. Across the unweighted and balanced analyses, all 500 outer folds selected exactly four features, correlation-cluster representative counts ranged from 8 to 12, and the training and test sets were participant-disjoint.

These results estimate a procedure that selects four features independently within each outer-training fold. They do not provide an unbiased validation of the fixed core-four set identified from the full-cohort analyses. The bootstrap intervals are conditional on the repeated OOF predictions and do not repeat splitting, selection, or fitting. Repetition also does not increase the effective sample size beyond 38 children. Detailed metrics, selection frequencies, participant-level split checks, and conditional bootstrap summaries are provided in the accompanying supplementary files.

S15b. Post hoc fixed four-feature analysis

The fixed candidate comprised EnvDuty50_mean, CCI_peaknorm_distal_mean, CCI_peaknorm_proximal_mean, and SpatialEntropy_mean. These descriptors were identified after the full-cohort association and repeated-CV analyses: they were the four most frequently selected features and also defined the principal association result. Because this outcome-informed selection used the complete development cohort, the selection step was not nested, and the internal metrics below are descriptive post-selection results rather than unbiased estimates for unseen individuals.

The fixed core-four and design-locked 16-pool analyses used the same participant-level outer partitions. Apparent repeat-level values were QWK 0.8894 ± 0.0209, MAE 0.1353 ± 0.0238, accuracy 0.8647 ± 0.0238, balanced accuracy 0.8718 ± 0.0273, and macro-F_1 0.8680 ± 0.0246. Recall was 0.920/0.789/0.906 for GMFCS I/II/III. Because they are descriptive post-selection estimates, these values are reported separately from the prespecified comparative performance results.

Across paired repeats, apparent fixed-core-four minus 16-pool differences were ΔQWK 0.0521±0.0444, ΔMAE −0.0605±0.0497, Δaccuracy 0.0605±0.0497, and Δmacro-F_1 0.0570±0.0485. Because the named set was selected after examining the development cohort, these differences do not establish superiority. The associated bootstrap intervals were conditional on the fixed post-selection specification and did not repeat feature selection.

S15c. Model-size selection and zero-selection-frequency features

Across 250 outer folds, the candidate grid was $\{3, 5, 8, 10\}$ and selected model sizes were $k = 3$ in 132 folds, $k = 5$ in 80, $k = 8$ in 30, and $k = 10$ in 8. Candidate sizes were restricted after training-fold correlation clustering so that k did not exceed the number of representative features available in any relevant training split. EnvDuty75_mean and EnvSpectralEntropy_mean were never selected (0/250); both features were evaluated within the design-locked pool and had zero selection frequency.

S16. Cross-Cohort Transportability and Domain-Shift Diagnostics

The locked transportability stress test fitted the all-16 or core-four model only on the 38 internal participants and applied it unchanged to the open-workbook participants. The in-domain reference instead refitted each specification within the open-workbook training folds and therefore measures within-dataset learnability.

The open-workbook sample had a different GMFCS prior (I/II/III = 55.4/29.7/14.9%) from the internal cohort (34.2/39.5/26.3%). Poor all-16 locked-transfer balanced accuracy (0.428) and macro-F_1 (0.266), together with prediction of only one participant as GMFCS I, indicated that transfer failure extended beyond differences in class prevalence. Core-four transfer was also poor: recall was 0.065, 0.172, and 0.862 for levels I, II, and III; 63/108 open-workbook GMFCS-I participants were assigned to level III. In the $n = 188$ sensitivity analysis, core-four QWK was 0.089, accuracy 0.218, and macro-F_1 0.207.

When the specifications were refitted within the open workbook, the fixed all-16 model yielded QWK 0.559±0.031 and the core-four candidate yielded 0.532±0.032 across 50 repeated five-fold evaluations. The corresponding balanced versions yielded QWK 0.576 ± 0.020 and 0.537 ± 0.022. These refitted estimates show that both representations retained GMFCS-related information in the open workbook; evaluation of the internal decision rules is provided by the locked-transfer analysis.

Repeated-CV logistic-regression and Extra-Trees domain classifiers both distinguished internal from open-workbook participants with ROC-AUC 1.000 and balanced accuracy 1.000. Thirteen of 16 feature–GMFCS associations retained their sign, but several were markedly attenuated in the open-workbook sample: SpatialEntropy_mean changed from $\rho = 0.852$ to 0.216, CCI_peaknorm_proximal_mean from 0.736 to 0.285, EnvCentroid_norm_mean from 0.653 to 0.216, and EnvSpectralEntropy_mean from 0.464 to 0.016. The three sign-discordant or near-null open-workbook relationships were burst duration (−0.445 versus 0.126), inter-muscle correlation (−0.090 versus 0.350), and peak-timing variability (−0.214 versus 0.004).

Using only the core four, logistic-regression and Extra-Trees domain classifiers still achieved ROC-AUC 0.977 and 0.976, with balanced accuracy 0.869 and 0.832. Core-feature Kolmogorov–Smirnov statistics were 0.777 for duty cycle, 0.095 for distal CCI, 0.358 for proximal CCI, and 0.266 for spatial entropy. All four association directions were concordant, but open-workbook associations were attenuated relative to internal values: duty cycle 0.683 to 0.482, distal CCI 0.751 to 0.393, proximal CCI 0.736 to 0.285, and spatial entropy 0.852 to 0.216.

The downstream CCI and envelope equations were shared, but their upstream inputs were not fully equivalent. Internal features began with raw Delsys sEMG and project-specific filtering, envelope construction, boundary-based cycle retention and resampling. Open-workbook features began with already filtered, rectified, normalized and concatenated activations and reconstructed 101-point cycle blocks. Internal single-cycle peak-normalized NMF descriptors were calculated from the internal sEMG data, whereas the open-workbook comparison used the deposited VAF quantities. The observed domain shift may reflect combined differences in device, preprocessing, cycle construction, anatomical mapping, and cohort composition.

Supplementary Tables

Table S1: Internal-cohort statistics for all 16 design-locked features. False discovery rate (FDR) correction was performed within the 16-feature descriptive family. Cliff's δ is I–II / II–III / I–III; negative values indicate larger values in the higher-GMFCS group. Selection frequency is selection in the 250 outer folds. Bootstrap confidence intervals are reported for the four core features in PDF Table S8a and for all 16 features in Supplementary Table S8b. CCI, co-activation index; GMFCS, Gross Motor Function Classification System; JT, Jonckheere–Terpstra; KW, Kruskal–Wallis; VAF, variance accounted for.

Feature	Selection	GMFCS I	GMFCS II	GMFCS III	ρ	Spearman q	KW q	JT q	Cliff's δ
EnvDuty50_mean	236/250 (94.4%)	0.157 ± 0.017	0.183 ± 0.046	0.274 ± 0.026	0.683	6.22×10^{-6}	1.12×10^{-4}	5.34×10^{-5}	-0.251/-0.920/-1.000
CCI_peaknorm_distal_mean	217/250 (86.8%)	0.601 ± 0.051	0.655 ± 0.053	0.733 ± 0.034	0.751	4.42×10^{-7}	1.12×10^{-4}	9.54×10^{-6}	-0.569/-0.827/-0.969
SpatialEntropy_mean	194/250 (77.6%)	0.785 ± 0.060	0.879 ± 0.055	0.922 ± 0.017	0.852	1.87×10^{-10}	2.35×10^{-5}	2.77×10^{-7}	-0.856/-0.813/-1.000
CCI_peaknorm_proximal_mean	157/250 (62.8%)	0.615 ± 0.036	0.645 ± 0.021	0.690 ± 0.035	0.736	6.36×10^{-7}	1.12×10^{-4}	9.54×10^{-6}	-0.569/-0.813/-0.938
EnvCentroid_norm_mean	89/250 (35.6%)	3.703 ± 0.156	3.912 ± 0.282	4.244 ± 0.235	0.653	2.05×10^{-5}	6.29×10^{-4}	1.05×10^{-4}	-0.446/-0.640/-0.923
EnvHL_ratio_high_over_low	65/250 (26.0%)	0.365 ± 0.056	0.470 ± 0.128	0.607 ± 0.113	0.701	3.06×10^{-6}	2.87×10^{-4}	3.93×10^{-5}	-0.538/-0.613/-0.985
SingleCyclePeakNorm_synergies_95VAF	48/250 (19.2%)	3.231 ± 0.599	2.933 ± 0.458	2.400 ± 0.699	-0.486	0.003	0.015	0.013	0.262/0.413/0.585
DistalProximal EnvelopeRatio	45/250 (18.0%)	0.677 ± 0.049	0.666 ± 0.049	0.621 ± 0.043	-0.460	0.005	0.015	0.007	0.179/0.600/0.677
EnvBurstDuration_mean	42/250 (16.8%)	0.028 ± 0.002	0.028 ± 0.002	0.026 ± 0.003	-0.445	0.006	0.017	0.011	0.128/0.520/0.723
SingleCyclePeakNorm_tVAF1	25/250 (10.0%)	0.817 ± 0.086	0.852 ± 0.041	0.905 ± 0.037	0.488	0.003	0.011	0.003	-0.231/-0.653/-0.677
EnvIntermuscleCorr_mean	15/250 (6.0%)	0.449 ± 0.074	0.470 ± 0.132	0.397 ± 0.142	-0.090	0.590	0.378	0.619	-0.179/0.307/0.200
EnvDuty25_mean	14/250 (5.6%)	0.350 ± 0.051	0.431 ± 0.105	0.611 ± 0.079	0.734	6.36×10^{-7}	1.12×10^{-4}	1.21×10^{-5}	-0.467/-0.840/-1.000
CCI_peaknorm_distal_proximal_ratio	7/250 (2.8%)	0.980 ± 0.102	1.018 ± 0.100	1.063 ± 0.058	0.425	0.009	0.038	0.013	-0.385/-0.227/-0.631
EnvPeakTiming_sd	5/250 (2.0%)	0.410 ± 0.073	0.392 ± 0.101	0.376 ± 0.072	-0.214	0.210	0.332	0.225	0.005/0.293/0.369
EnvDuty75_mean	0/250 (0.0%)	0.084 ± 0.005	0.090 ± 0.014	0.113 ± 0.009	0.624	5.63×10^{-5}	3.61×10^{-4}	3.49×10^{-4}	-0.159/-0.787/-1.000

Feature	Selection	GMFCS I	GMFCS II	GMFCS III	ρ	Spearman q	KW q	JT q	Cliff's δ
EnvSpectralEntropy_ mean	0/250 (0.0%)	0.847 ± 0.022	0.856 ± 0.038	0.889 ± 0.028	0.464	0.005	0.015	0.009	-0.138/-0.507/-0.769

Table S2: Directional evaluation of harmonized counterparts of the 16 features in 195 unique participants from the open workbook (Table S1 accompanying Goudriaan et al.). Sample inclusion and the $n = 188$ sensitivity analysis are described in Section S12. Several descriptors used approximate muscle mapping or table-derived NMF quantities and are not exact reproductions. Absolute values are cohort- and preprocessing-specific; inference concerns within-workbook GMFCS trends. All three q-value columns report Benjamini–Hochberg adjustment across the 16 features within the corresponding test family. GMFCS, Gross Motor Function Classification System; NMF, non-negative matrix factorization.

Feature	GMFCS I	GMFCS II	GMFCS III	ρ	Spearman q	KW q	JT q
CCI_peaknorm_distal_ mean	0.641 ± 0.067	0.679 ± 0.069	0.726 ± 0.059	0.393	3.54×10^{-8}	2.75×10^{-7}	1.14×10^{-7}
CCI_peaknorm_distal_ proximal_ratio	0.994 ± 0.170	1.008 ± 0.197	1.166 ± 0.338	0.092	0.232	0.212	0.236
CCI_peaknorm_proximal_ mean	0.654 ± 0.077	0.687 ± 0.088	0.665 ± 0.158	0.285	9.63×10^{-5}	6.31×10^{-4}	5.96×10^{-5}
DistalProximalEnvelopeRatio	0.667 ± 0.081	0.658 ± 0.085	0.624 ± 0.092	-0.146	0.056	0.089	0.056
EnvBurstDuration_mean	0.127 ± 0.017	0.133 ± 0.021	0.130 ± 0.029	0.126	0.098	0.212	0.093
EnvCentroid_norm_mean	2.832 ± 0.286	2.851 ± 0.322	3.153 ± 0.443	0.216	0.004	0.001	0.004
EnvDuty25_mean	0.590 ± 0.098	0.657 ± 0.095	0.724 ± 0.115	0.427	2.02×10^{-9}	7.09×10^{-8}	6.68×10^{-9}
EnvDuty50_mean	0.328 ± 0.057	0.376 ± 0.075	0.441 ± 0.078	0.482	7.95×10^{-12}	6.52×10^{-10}	5.88×10^{-11}
EnvDuty75_mean	0.160 ± 0.025	0.176 ± 0.032	0.195 ± 0.032	0.400	2.19×10^{-8}	2.75×10^{-7}	6.11×10^{-8}
EnvHL_ratio_high_over_ low	0.170 ± 0.098	0.188 ± 0.096	0.320 ± 0.150	0.333	3.88×10^{-6}	9.49×10^{-7}	5.92×10^{-6}
EnvIntermuscleCorr_mean	0.209 ± 0.118	0.273 ± 0.116	0.333 ± 0.167	0.350	1.19×10^{-6}	1.36×10^{-5}	1.51×10^{-6}
EnvPeakTiming_sd	0.328 ± 0.038	0.333 ± 0.049	0.317 ± 0.064	0.004	0.960	0.591	0.931
EnvSpectralEntropy_mean	0.664 ± 0.044	0.654 ± 0.053	0.673 ± 0.063	0.016	0.874	0.515	0.864
SingleCyclePeakNorm_ synergies_95VAF	4.167 ± 0.588	3.862 ± 0.687	3.241 ± 0.511	-0.442	5.14×10^{-10}	1.02×10^{-9}	1.43×10^{-7}

Feature	GMFCS I	GMFCS II	GMFCS III	ρ	Spearman q	KW q	JT q
SingleCyclePeakNorm_tVAF1	0.748 ± 0.059	0.798 ± 0.057	0.859 ± 0.029	0.586	3.36×10^{-18}	6.00×10^{-15}	5.34×10^{-15}
SpatialEntropy_mean	0.895 ± 0.038	0.911 ± 0.036	0.897 ± 0.066	0.216	0.004	0.010	0.002

Table S3: Gait-cycle boundary detection and quality-control summary by GMFCS level. Values are participant-level median [range] unless otherwise stated. GMFCS, Gross Motor Function Classification System; LG, lateral gastrocnemius; SD, standard deviation; SO, soleus; TA, tibialis anterior.

	GMFCS I ($n = 13$)	GMFCS II ($n = 15$)	GMFCS III ($n = 10$)
Detected boundaries	50 [28–65]	38 [26–53]	46 [35–58]
Initial cycles	49 [27–64]	37 [25–52]	45 [34–57]
Retained cycles	43 [27–61]	37 [23–52]	43.5 [32–56]
Removed fraction	0.034 [0–0.204]	0 [0–0.080]	0.035 [0–0.135]
Cycle-duration coefficient of variation, mean ± SD (%)	11.50 ± 4.73	13.66 ± 6.65	16.97 ± 4.62
TA/LG/SO boundary source, n	13	15	10

Table S4: Summary of feature-definition sensitivity analyses. The largest JT q is the least favorable false-discovery-rate-adjusted Jonckheere–Terpstra value across variants; weakest $|\delta|$ is the smallest absolute GMFCS I-versus-III Cliff's effect. GMFCS, Gross Motor Function Classification System; JT, Jonckheere–Terpstra.

Core feature	Spearman ρ range	Largest JT q	Weakest $\lvert\delta\rvert$
Activation duty cycle	0.446–0.780	0.00942	0.754
Distal peak-normalized CCI	0.620–0.778	8.09×10^{-5}	0.846
Spatial entropy	0.764–0.874	2.09×10^{-6}	0.938
Proximal peak-normalized CCI	0.534–0.765	0.000808	0.754

Supplementary Table S5 (machine-readable). Complete feature-definition sensitivity results for all 84 feature–variant evaluations are provided in Supplementary_Table_S5_feature_definition_sensitivity_84_evaluations.csv.

Supplementary Table S6 (machine-readable). Internal- and open-workbook feature-direction concordance results are provided in Supplementary_Table_S6_internal_open_workbook_direction_concordance.csv.

Table S7: Additional post hoc model-family benchmarks under the same 50×5 outer partitions used for the design-locked 16-feature analysis. These additional model-family analyses were post hoc and are reported as contextual benchmarks. QWK is reported as mean ± standard deviation across repeats; MAE, accuracy, macro-F_1, and class-specific recall are repeat-averaged values. These summaries describe resampling variability rather than independent-sample uncertainty. LDA, linear discriminant analysis; MAE, mean absolute error; QWK, quadratic weighted kappa.

Model	QWK	MAE	Accuracy	Macro-F_1	Recall I/II/III
k-nearest neighbors ($k = 5$)	0.835 ± 0.040	0.199	0.801	0.808	0.818/0.736/0.876
Shrinkage LDA	0.820 ± 0.029	0.218	0.782	0.790	0.720/0.721/0.954
Histogram gradient boosting	0.789 ± 0.064	0.246	0.756	0.763	0.743/0.715/0.834
Gaussian Naive Bayes	0.783 ± 0.054	0.251	0.749	0.759	0.714/0.728/0.826
Gradient boosting	0.776 ± 0.051	0.262	0.739	0.746	0.760/0.697/0.774
AdaBoost	0.776 ± 0.048	0.267	0.733	0.737	0.795/0.665/0.752
Decision tree	0.744 ± 0.051	0.289	0.712	0.715	0.769/0.700/0.656

Table S8a: Participant-level stratified percentile-bootstrap 95% confidence intervals for the four core feature effect sizes (B = 10,000; seed 20260724). Cliff's δ uses the GMFCS I-minus-III convention; negative values indicate larger values in GMFCS III. Supplementary Table S8b reports all 16 design-locked features. CCI, co-activation index; CI, confidence interval; GMFCS, Gross Motor Function Classification System.

Feature	Spearman ρ	95% CI	Cliff's δ I–III	95% CI
Activation duty cycle	0.683	0.549–0.811	−1.000	−1.000–−1.000
Distal peak-normalized CCI	0.751	0.592–0.883	−0.969	−1.000–−0.862
Spatial entropy	0.852	0.751–0.927	−1.000	−1.000–−1.000
Proximal peak-normalized CCI	0.736	0.571–0.864	−0.938	−1.000–−0.769

Supplementary Table S8b (machine-readable). Bootstrap confidence intervals for effect sizes of all 16 design-locked features are provided in Supplementary_Table_S8b_effect_size_bootstrap_ci_all16.csv.

Table S9a: Locked transfer and diagnostic alignment analyses in the 195-participant open-workbook sample. Alignment used the complete unlabeled open-workbook marginal distribution as a post hoc diagnostic analysis. IQR, interquartile range; MAE, mean absolute error; QWK, quadratic weighted kappa.

Analysis	QWK	MAE	Accuracy	Balanced accuracy	Macro-F_1
Locked internal→open-workbook transfer	0.210	0.836	0.277	0.428	0.266
Median/IQR alignment	0.426	0.538	0.497	0.541	0.508
Marginal quantile alignment	0.436	0.559	0.482	0.535	0.493
Open-workbook→internal reverse transfer	0.078	0.868	0.368	0.367	0.234
Core-four internal→open-workbook transfer	0.077	1.128	0.215	0.366	0.203

Table S9b: Largest marginal shifts among the 16 harmonized features. D is the two-sample Kolmogorov–Smirnov statistic. Fourteen of 16 features remained different after Benjamini–Hochberg correction, and 11/16 had $D \geq 0.50$. KS, Kolmogorov–Smirnov.

Feature	Internal median	Open-workbook median	KS D
EnvBurstDuration_mean	0.0273	0.1274	1.000
EnvSpectralEntropy_mean	0.8562	0.6651	0.995
EnvDuty75_mean	0.0884	0.1665	0.954
EnvCentroid_norm_mean	3.8658	2.8307	0.938
EnvHL_ratio_high_over_low	0.4426	0.1690	0.840
EnvDuty50_mean	0.1735	0.3472	0.777
EnvPeakTiming_sd	0.4275	0.3325	0.653
SingleCyclePeakNorm_synergies_95VAF	3.000	4.000	0.633

Supplementary Table S10 (machine-readable). Feature-level recording-endpoint cycle-exclusion sensitivity results are provided in Supplementary_Table_S10_recording_endpoint_cycle_sensitivity.csv.
